# Piperazine‐Functionalized Nanoparticles Enable Oral Insulin Delivery in Obese Mice

**DOI:** 10.1002/advs.202520918

**Published:** 2026-03-07

**Authors:** Yuxue Cao, Xiaofan Jiang, Md Moniruzzaman, Alexandra Mueller, Taskeen Iqbal Janjua, Nisha Tyagi, Zhi Qu, Kuan Yau Wong, Yuran Feng, Aayushi Ghodasara, Ekaterina Strounina, Benjamin P. Ross, Tushar Kumeria, Sumaira Z. Hasnain, Amirali Popat

**Affiliations:** ^1^ School of Pharmacy and Pharmaceutical Sciences The University of Queensland Brisbane QLD Australia; ^2^ Drug Delivery, Disposition and Dynamics Monash Institute of Pharmaceutical Sciences Monash University Parkville VIC Australia; ^3^ Faculty of Medicine The University of Queensland Woolloongabba QLD Australia; ^4^ Immunopathology Group Mater Research Institute Translational Research Institute The University of Queensland Brisbane QLD Australia; ^5^ Centre for Advanced Imaging The University of Queensland QLD Australia; ^6^ School of Materials Science and Engineering The University of New South Wales Sydney NSW Australia; ^7^ Australian Centre For Nanomedicine The University of New South Wales Sydney NSW Australia; ^8^ Australian Infectious Diseases Research Centre The University of Queensland Brisbane QLD Australia; ^9^ A Department of Functional Materials and Catalysis Faculty of Chemistry University of Vienna Vienna Austria

**Keywords:** drug delivery, insulin, oral delivery, permeation enhancer, pharmaceutical sciences, silica nanoparticles, 1‐phenylpiperazine

## Abstract

Biologics have gained prominence as a rapidly advancing therapeutic modality for a diverse spectrum of medical conditions. Nonetheless, they are predominantly administered parenterally due to poor absorption through the gastrointestinal tract. Additionally, repeated injections of biologics such as insulin cause injection pain, leading to dose‐skipping and poor medication adherence. Recently, our group and others have shown that nanoparticle‐based drug delivery systems enhance intestinal permeation and oral delivery of biologics such as insulin and exenatide. However, their effectiveness is not on par with chemical permeation enhancers (PEs) including  fatty acids, lipids, and 1‐phenylpiperazine (PPZ), which are toxic at their effective doses. In this work, we report successful PPZ grafting onto silica nanoparticles with no apparent toxicity and significantly increased permeation of macromolecules such as insulin in an in vitro Caco‐2 monolayer model and a co‐culture model. In vivo experiments, with silica‐PPZ‐based novel PE improves the permeation of FITC‐dextran‐4 kDa in healthy mice compared to bare silica nanoparticles and macromolecule alone. In a high‐fat diet mouse model, the use of silica‐PPZ significantly enhances insulin absorption in the intestine. This led to markedly lower and more sustained blood glucose levels compared to controls without any associated toxicity to mice. Overall, we have shown, for the first time, that PPZ‐grafted silica nanoparticles can serve as a safe and effective permeation enhancer for the oral administration of insulin and potentially other biologics.

## 1 Introduction

With the exponential rise in diabetes, the numbers have reached pandemic levels, and the disease is one of the leading causes of mortality worldwide [1]. Insulin, administered via injections or pumps, is recommended in 100% of the Type 1 Diabetic patients and approximately 25% of Type 2 Diabetes cases [2, 3]. Fear of needles is a very common and often debilitating phobia that affects people of all ages, especially children and young adults [3]. As a result, around 30% of diabetic patients report feeling dread associated with insulin injection, and close to 50% have reported to have intentionally skipped their doses [4]. Therefore, oral insulin delivery would have enormous benefits and would markedly improve patient experience, adherence, and disease outcomes [5]. However, oral administration of macromolecule therapeutics such as insulin has always been a challenge due to their poor oral bioavailability into the bloodstream because of poor permeation of the intestinal epithelium and mucus barriers. Several strategies, such as nanoparticles, ionic liquids, and chemical permeation enhancers (PEs), have been utilized to overcome the intestinal barriers to oral insulin delivery [6, 7, 8]. However, no formulation has successfully cleared all clinical hurdles, and therefore, no oral insulin products are commercially available. Currently, one of the most effective strategies to orally deliver biologics is via the use of the permeation enhancers salcaprozate sodium (SNAC) and sodium caprate (C10). PEs are excipients that have been added to many oral formulations to increase the oral bioavailability of drugs by enhancing intestinal absorption [9]. Traditional PEs primarily include detergents, surfactants, fatty acids, and Ca^2+^‐chelating agents. These PEs are believed to facilitate intestinal absorption by increasing the paracellular permeability of epithelial cells through intracellular signalling pathways or direct disruption of homophilic interactions at cell adhesion recognition (CAR) sequences [10]. For example, SNAC and C10 played an important role in the oral absorption of semaglutide (Rybelsus, Novo Nordisk, 2019), which was the first FDA‐approved oral glucagon‐like peptide 1 (GLP‐1) receptor agonist that manages Type 2 diabetes [11, 12]. In 2020, an oral formulation of octreotide (MYCAPSSA capsule) applied transient permeability enhancer (TPE) technology to enhance the permeability of the drug across the mucus and intestinal epithelial barriers by transiently altering epithelial barrier integrity, using sodium C10 as PE [13, 14]. However, disadvantages of PEs include irritation and depletion or binding of trace elements, which leads to nutritional deficiency [9, 15]. Additionally, patient adherence is an issue with Rybelsus since patients need to wait for at least 30 min after taking medication before food intake. Thus, there is an urgent need for safe and effective PEs to improve the bioavailability of oral macromolecule drugs is still a challenging task.

In early 2008, 1‐phenylpiperazine (PPZ) emerged as a chemical permeation enhancer, where PPZ could significantly increase the permeability of hydrophilic marker molecules, mannitol, and macromolecule (70 kDa) dextran in an in vitro Caco‐2 monolayer model [16]. Subsequently, the efficacy and toxicity of PPZ were investigated ex vivo using rat ileal and colonic mucosa in a Ussing chamber [17]. The results indicated that PPZ promoted mannitol transport across the Caco‐2 monolayers and rat ileal and distal colonic mucosa models at the concentration of 0.6 – 60 mm. It induced tight junction opening through intracellular mechanisms involving the modulation of myosin light chain kinase. Notably, PPZ concentrations up to 6 mm did not cause significant cellular or tissue damage, but higher concentrations showed significant tissue damage in ex vivo study [17]. In addition to modulation of myosin light chain kinases, PPZ also exerts its effects by activating 5‐HT_4_ receptors, which regulate gastrointestinal physiology, and the basolateral Na^+^/K^+^/2Cl^−^ cotransporter which is associated with electrogenic chloride secretion [18]. Despite the promising efficacy of PPZ, its toxicity limits its therapeutic application at concentrations above 6 mM. Therefore, developing safer delivery strategies and optimising dosing regimens are crucial for maximising the therapeutic potential of PPZ [19].

Our laboratory and others have recently discovered that silica nanoparticles improve the permeability of peptides and proteins by transiently opening the intestinal epithelial tight junctions to increase protein absorption and bioavailability [4, 20, 21, 22, 23, 24, 25]. Silica nanoparticles have emerged as a versatile drug delivery system due to their tunable properties and biocompatibility [26]. Their ability to be synthesized with desired size, shape, and pore structure facilitates their tailoring to specific drug delivery applications [27]. Moreover, the high surface area of mesoporous silica nanoparticles allows easy surface modification [28]. Importantly, silica nanoparticles are generally recognized as a safe material, with a history of successfully cleared Phase I trials in biomedical applications [26]. Recently, Lamson et al. found that small‐sized (< 200 nm) anionic silica nanoparticles could also enhance intestinal permeability of insulin via myosin light chain kinase to open the tight junctions [25]. Oral administration of silica nanoparticles followed by insulin capsules induced sustained blood glucose control in mice with diabetes [25]. Inspired by these results, we hypothesized that PPZ grafted onto silica nanoparticles could further improve its permeation enhancement ability and, more importantly, reduce cytotoxicity. Therefore, here we functionalized PPZ onto silica nanoparticles and investigated its ability to enhance the intestinal permeation of multiple macromolecules in vitro and in vivo.

## 2 Results and Discussion

### 2.1 Synthesis of 1‐Phenyl‐4‐[3‐(trimethoxysilyl)propyl]piperazine

**FIGURE 1:**
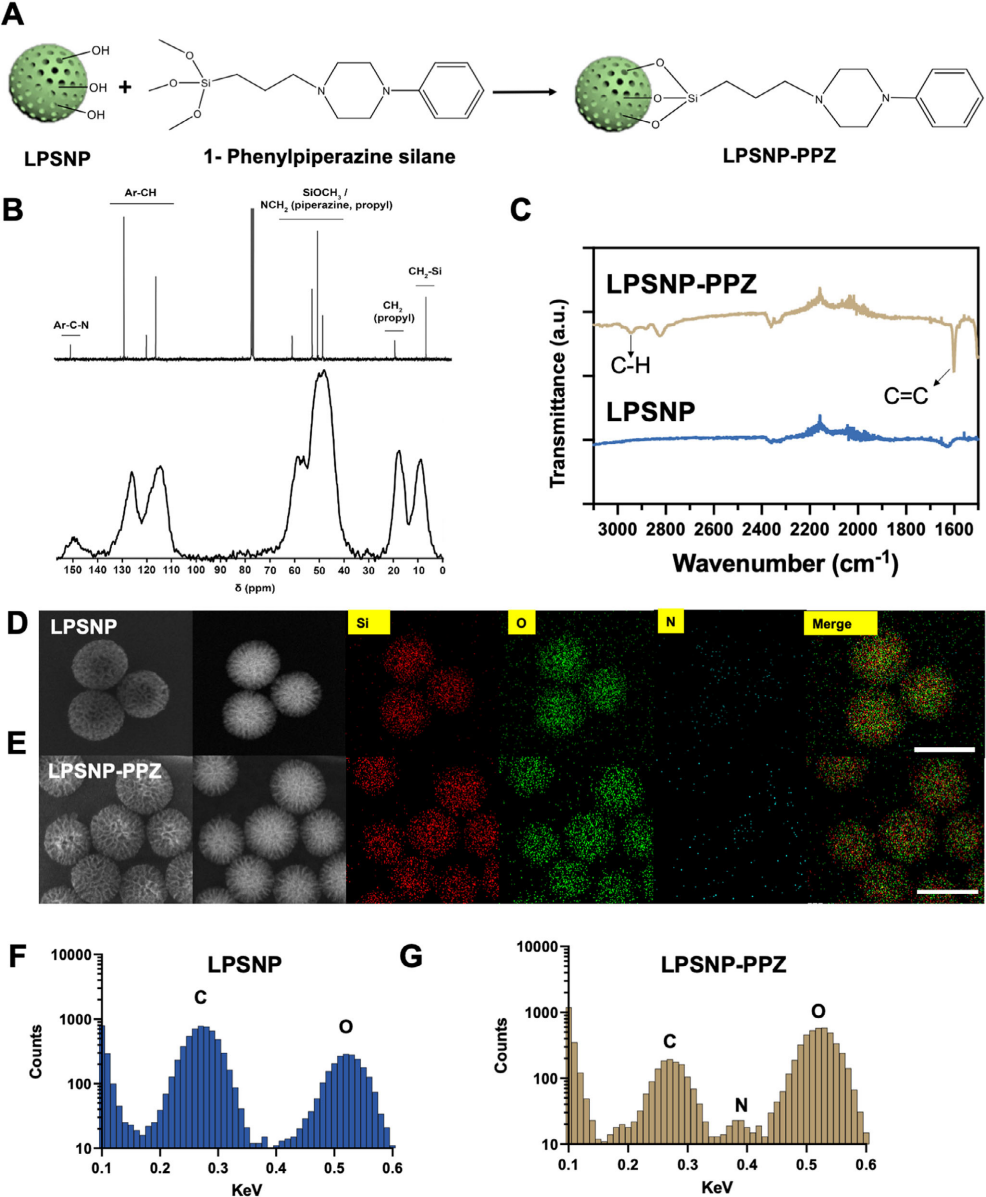
(A) Scheme showing grafting of 1‐phenyl‐4‐[3‐(trimethoxysilyl)propyl]piperazine (“PPZ silane”) to LPSNP to generate LPSNP‐1‐phenylpiperazine (LPSNP‐PPZ). (B) ^1^ ^3^C NMR spectrum of PPZ silane in CDCl_3_ solution (75 MHz, top) and solid‐state ^1^ ^3^C NMR spectrum of the grafted material (LPSNP‐PPZ, bottom). Horizontal grouping lines indicate functional‐group‐level assignments based on characteristic chemical‐shift ranges for analogous silanes. (C) FTIR spectra of LPSNP and LPSNP‐PPZ nanoparticles in the wavenumber range 1500–3100 cm^−1^. (D) SEM, dark field STEM, and elemental analysis of LPSNP. (E) SEM, dark field STEM, and elemental analysis of LPSNP‐PPZ. Scale bar: 100 nm. (F), (G) Elemental scan of LPSNP and LPSNP‐PPZ.

1‐Phenyl‐4‐[3‐(trimethoxysilyl)propyl]piperazine for silanization of large pore silica nanoparticles (LPSNP) was synthesized by reacting (3‐iodopropyl)trimethoxysilane with PPZ in the presence of triethylamine and under an atmosphere of nitrogen, while stirring (Figure S1). After purification by vacuum distillation, the identity of the product was confirmed to be 1‐phenyl‐4‐[3‐(trimethoxysilyl)propyl]piperazine by analysis using ^13^C NMR and ^1^H NMR spectroscopy. Ten distinct resonances were observed in the ^1^ ^3^C NMR spectrum (Figure 1B, top, and Figure S2). At the functional‐group level, these can be grouped as follows: Ar–C–N and Ar–CH carbons at 151.0–116.5 ppm; SiOCH_3_ / NCH_2_ (piperazine, propyl) carbons in the 61.0–48.7 ppm region; CH_2_ (propyl) at 19.4 ppm; and CH_2_–Si at 6.8 ppm. The ^1^H NMR spectrum (Figure S3) contained resonances for the 5 aromatic protons (Ar–H) between 7.3 and 6.8 ppm, the 9 protons of the three SiOCH_3_ groups at 3.6 ppm, the 8 piperazine NCH_2_ protons between 3.4 and 2.6 ppm, and the 6 protons of the three CH_2_ groups of the propyl unit between 2.6 and 0.6 ppm. Overall, the NMR data confirmed successful synthesis of 1‐phenyl‐4‐[3‐(trimethoxysilyl)propyl]piperazine (also referred to in this manuscript as “PPZ silane”), with no apparent impurities following vacuum distillation.

### 2.2 Synthesis and Characterization of 1‐Phenylpiperazine Functionalized LPSNP

LPSNP were synthesized, using cetrimonium chloride (CTAC) as a template, triethanolamine (TEA) as a catalyst, and tetraethyl orthosilicate (TEOS) as a silica precursor [29]. The surface of LPSNP was modified by silanization with PPZ silane, as shown in Figure 1A, modified from our published silane coupling method [28, 30]. After functionalization, the nanoparticles, LPSNP‐1‐phenylpiperazine (LPSNP‐PPZ), were analysed by solid‐state NMR (^13^C and ^29^Si), FTIR, and elemental analysis. The pattern observed in the solid‐state ^1^ ^3^C NMR spectrum of the grafted material (Figure 1B, bottom) was highly consistent with that of the ^1^ ^3^C NMR spectrum of the PPZ silane in solution (Figure 1B, top). Although the solid‐state resonances are substantially broadened, they can be grouped into the same chemical‐shift regions as for the solution spectrum, corresponding to Ar–C–N and Ar–CH carbons, SiOCH_3_ / NCH_2_ (piperazine, propyl) carbons, CH_2_ (propyl), and CH_2_–Si. This close correspondence in chemical‐shift distribution, together with the absence of additional resonances of comparable intensity, supports that the PPZ silane retains its expected structure after grafting to the nanoparticle surface. The solid‐state ^29^Si NMR spectrum is shown in Figure S4, and the relative peak areas are listed in Table S1. LPSNP exhibited 3 peaks assigned to Q2 (Si(OSi)_2_(OH)_2_, −94.49 ppm), Q3 (Si(OSi)_3_OH, −106.32 ppm), and Q4 (Si(OSi)_4_, −114.81 ppm) silicon environments [31, 32]. After functionalization, T2 [(OH)(SiO)_2_Si–R] and T3 [(SiO)_3_Si–R] peaks were detected at −63.56 and −71.91 ppm, consistent with grafting of PPZ silane onto the silica surface [33]. Overall, these data indicate successful surface functionalization of LPSNP.

FTIR and elemental analysis were further conducted to confirm the grafting of PPZ silane to LPSNP. In FTIR (Figure S5), both LPSNP and LPSNP‐PPZ showed a broad peak at 1100 cm^−1^ indicating the Si‐O‐Si stretching vibration, and the peak at 460 cm^−1^ corresponding to the Si‐O bond stretching [34]. However, in the enlarged FTIR spectrum (Figure 1C, 1500–3100 cm^−1^), LPSNP‐PPZ showed a peak at 1600 cm^−1^, which links to the C = C stretching. While the peaks around 3000 cm^−1^ are linked to the C‐H bonds. Figure 1D, E showed the SEM, dark field scanning transmission electron microscopy (STEM), and elemental analysis of LPSNP and LPSNP‐PPZ, respectively. From the SEM and STEM, the large pore and dendritic structure were shown in the Figure 1D, E. The elemental analysis (Figure 1F, G) proved the existence of Si and O in both particles, and a trace amount of N signal was captured in LPSNP‐PPZ and further proved by the mapping (Figure 1G), which indicated the N on the piperazine ring. C was detected in both samples as the nanoparticles were placed on a carbon film‐coated grid for imaging. In addition to the qualitative elemental mapping obtained by STEM–EDS, we further performed CHN combustion analysis to quantify the nitrogen introduced by PPZ functionalization (Table S2). These findings are consistent with the nitrogen signals observed in STEM–EDS. These results provide further confirmation that the PPZ silane was successfully grafted to LPSNP.

Distinct surface grafting densities were achieved by silanization of LPSNP using different amounts of PPZ silane, resulting in high (LPSNP‐PPZ_H_), medium (LPSNP‐PPZ_M_), and low (LPSNP‐PPZ_L_). LPSNP‐PPZ_H_ exhibited increased hydrophobicity (Figure S6A). As observed in centrifuge tube 2, LPSNP‐PPZ_H_ floated on the water's surface, while unmodified LPSNP remained well dispersed throughout the aqueous phase (centrifuge tube 1). LPSNP‐PPZ_M_ settled at the bottom of the centrifuge tube (tube 3), while LPSNP‐PPZ_L_ remained well dispersed in water (tube 4). Contact angle measurements further confirmed the differences in hydrophilicity (Figure S6B–F). LPSNP, LPSNP‐PPZ_H_, LPSNP‐PPZ_M_, and LPSNP‐PPZ_L_ identified a contact angle at 61.2°, 135.6°, 67.6°, and 22.9°, respectively. The results suggested that high‐density grafting of PPZ increased hydrophobicity, with a contact angle over than 90° indicated poor wetting ability [35]. However, with low‐density grafting of PPZ or pristine LPSNP, the contact angles were below 90°, which suggested good wetting ability. The LPSNP‐PPZ_L_ showed the lowest contact angle might be contributed by the higher hydroxyl group on the surface of nanoparticles which generated during the grafting process. As shown in Figure S7, LPSNP‐PPZ_L_ indicated stronger hydroxyl group stretching at around 3500 cm^−1^ [36, 37]. The hydrophilicity of nanoparticles could be an important factor that impacts cell interaction [38] or cytotoxicity, especially for the intestinal epithelial cells like Caco‐2 cells [39, 40].

**FIGURE 2:**
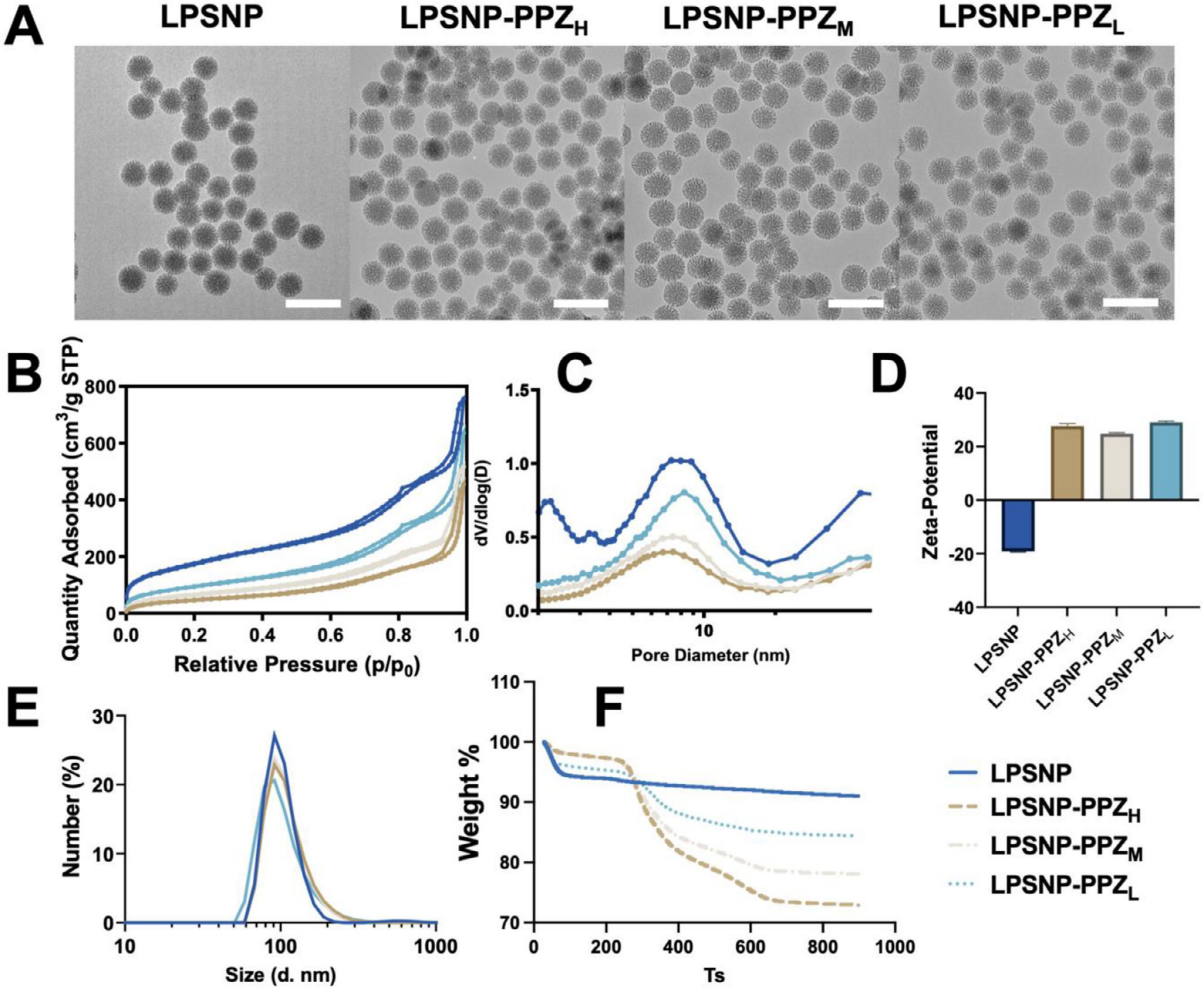
Characterization of LPSNP, LPSNP‐PPZ_H_, LPSNP‐PPZ_M,_ and LPSNP‐PPZ_L_. (A) TEM images, scale bar: 200 nm. (B) N_2_ physisorption isotherms, (C) corresponding to the BJH (Barrett–Joyner–Halenda) pore size distribution. (D) Zeta potential *n* = 3, mean ± SD (E) DLS analysis of nanoparticles suspensions in Milli‐Q water. (F) TGA weight loss of nanoparticles from 25 to 900°C.

**FIGURE 3:**
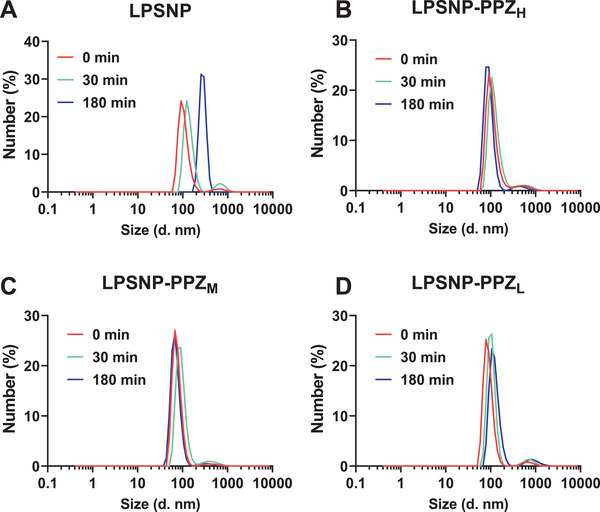
Representative hydrodynamic particle sizes measured by DLS after exposure to 10 mg/mL Type III mucus originating from porcine for (A) LPSNP, (B) LPSNP‐PPZ_H_, (C) LPSNP‐PPZ_M_, (D) LPSNP‐PPZ_L_.

Transmission electron microscopy (TEM) for the LPSNP and PPZ functionalized nanoparticles revealed that the particle size of LPSNP was approximately 90 nm, exhibiting a distinct dendritic structure (Table 1; Figure 2A). Functionalization with different densities of PPZ did not significantly alter the structure and size of the nanoparticles. Their specific surface area, pore volume and size, and pore size distribution of pristine and functionalized LPSNP were measured by nitrogen sorption and shown in Figure 2B, C, and summarised in Table 1. All the particles showed a typical type IV isotherm indicating the mesoporous structure (Figure 2B) consistent with the literature [41]. The pristine LPSNP demonstrated the highest BET surface area (634 m^2^/g). The surface area gradually reduced with increasing PPZ grafting density, resulting in 348 m^2^/g, 241 m^2^/g, and 173 m^2^/g for LPSNP‐PPZ_L,_ LPSNP‐PPZ_M_, and LPSNP‐PPZ_H_, respectively. This trend was mirrored by the pore size (Figure 2C) and pore volume, with pristine LPSNP exhibiting a pore diameter of 8.8 nm and 1.17 cm^3^/g, while functionalized particles (i.e., those bearing PPZ groups) displayed reduced pore size between 8.2 to 7.4 nm and 0.97 to 0.70 cm^3^/g. This is attributed to the PPZ grafting onto the surface of and within the pores of silica nanoparticles, leading to a reduction in surface area and pore size. Zeta‐potential measurements revealed a negative charge for pristine LPSNP due to the presence of silanol groups (Figure 2D) [37]. After the coupling of the PPZ group, the particles shifted to a positive charge at around +25 to +30 mV. The DLS measures the hydrodynamic particle size, which includes the particle and its surrounding water molecules. Therefore, the average size measured by DLS was higher than the actual particle size measured by TEM, ranging from 150 to 200 nm [42] (Figure 2E). In our series, the increase in hydrophobic PPZ content systematically reduces the difference between TEM and DLS diameters. This suggests that more hydrophobic surfaces bind a thinner, less structured hydration shell and likely promote a more compact conformation of the polymeric corona, leading to a smaller hydrodynamic diameter despite similar TEM core sizes [43]. The PDI ranging from 0.15 to 0.35 indicated the particles were well dispersed in the aqueous phase. The TGA measured the weight loss of the nanoparticles upon heating from 25°C to 900°C (Table S3; Figure 3F). The weight loss observed below 150°C, indicative of physically adsorbed water, was 5.9%, 4.4%, 2.4%, and 2.3% for LPSNP, LPSNP‐PPZ_L_, LPSNP‐PPZ_M_, and LPSNP‐PPZ_H_, respectively. This trend of decreasing water content was also attributed to increasing surface hydrophobicity due to higher grafting densities of PPZ groups. Organic content was estimated by excluding weight loss attributable to physically adsorbed water (below 150°C) and calculating the net weight loss between 150°C and 900°C, corrected by subtracting the corresponding weight loss of the unfunctionalized particles (LPSNP) (i.e., particles prior to grafting PPZ). The measured organic content (wt %) was used to calculate the grafting density (mmol of PPZ groups per gram of particles), and, together with the BET surface area of the unfunctionalized silica, the surface coverage (molecules per nm^2^) (Table S3). The results demonstrated the ability to control the grafting density and surface coverage of PPZ groups by varying the amount of PPZ silane used in the silanization of LPSNP. Overall, these comprehensive characterisations confirm the successful grafting of LPSNP with varying densities of the PPZ group, while maintaining comparable particle size and pore size.

**TABLE 1:** Physicochemical parameters of the pure and the functionalized silica materials, mean ± SD.

	Z‐Ave (d. nm)	PDI	Size TEM (d. nm)	Zeta‐potential (mV)	BET surface area (m^2^/g)	Pore size (nm)	Pore volume (cm^3^/g)
LPSNP	181.2 ± 2.6	0.28 ± 0.03	90.5 ± 6.4	−19.8 ± 0.4	634	8.8	1.17
LPSNP‐PPZ_L_	205.3 ± 5.4	0.34 ± 0.01	92.9 ± 7.5	29.0 ± 0.5	348	8.2	0.97
LPSNP‐PPZ_M_	149.4 ± 0.6	0.15 ± 0.01	93.9 ± 6.4	24.8 ± 0.3	241	7.4	0.80
LPSNP‐PPZ_H_	148.8 ± 0.3	0.15 ± 0.01	93.1 ± 6.3	27.6 ± 0.9	174	7.4	0.70

### 2.3 Mucin Interaction with PPZ Particles

The particle‐mucin interaction was evaluated using DLS at various time intervals following the dispersion of silica nanoparticles in mucin solution [25, 44]. Mucus is a complex dynamic hydrogel, composed mainly of cross‐linked and entangled mucin fibres, ranging in size from 0.5 to 40 MDa, and additionally containing other proteins, carbohydrates, lipids, bacteria, and cellular debris [45, 46]. Mucus also serves as a barrier, impeding the access of various substances to underlying tissues and the bloodstream [44]. Nanoparticles that become entrapped by mucus may experience an increase in particle size, making it challenging for them to penetrate through the mucus barrier. The data revealed that LPSNP increased in size from around 90 to 255 nm after 180 min incubation period (Figure 3A). While the PPZ functionalized particles maintained the size at around 100 nm throughout the 180 min interaction (Figure 3B–D). These findings suggest that PPZ functionalization might be able to reduce the interaction with mucus, potentially facilitating their passage across the mucus layer to reach the epithelial cells.

The mucin co‐incubation assay represents a simplified model that primarily reports on direct nanoparticle–mucin interactions and does not fully capture the structural and compositional differences between dense, acidic gastric mucus and the more porous, near‐neutral intestinal mucus. Nevertheless, porcine gastric mucin is widely used as an in vitro surrogate to assess nanoparticle–mucin interactions and relative trends in mucus penetration [47]. Within this limitation, the comparatively modest increase in hydrodynamic size observed for the hydrophobic, piperazine‐functionalized LPSNP‐PPZ_H_ relative to anionic unmodified particles suggests that charge‐distributed, dendritic cationic surfaces engage mucin in a more localized and transient manner, avoiding extensive corona formation and bridging aggregation that would otherwise strongly hinder diffusion. In contrast to conventional uniformly cationic coatings that often promote mucus trapping, this behavior indicates that the effective mucoadhesion of LPSNP‐PPZ_H_ is moderated despite their positive charge, which may help reconcile their in vitro mucus interaction. Interestingly, Iriarte‐Mesa et al. [48] showed that surface chemistry critically controls nanoparticle mobility in the same hybrid mucus–epithelium model, with formulations that minimize strong electrostatic and hydrophobic binding to mucins exhibiting more rapid translocation across the mucus layer. In that context, LPSNP‐PPZ_H_ occupies an interesting position that the dense piperazine functionalization appears to modulate these interactions so that particles are not irreversibly trapped by mucus.

To evaluate how PPZ surface modification affects nanoparticle transport across intestinal mucus, we quantified the apparent permeability (Papp) for particle diffusion over time (Figure S8). At 0.5 h, all LPSNP formulations displayed relatively high mucus permeability, with LPSNP–PPZ_H_ showing a modest but significant increase in Papp compared with unmodified LPSNP. After 1 h, permeability decreased for all groups, and no significant differences were detected between formulations. By 3 h, Papp further declined, and unmodified LPSNP retained significantly higher permeability than each PPZ‐modified formulation, indicating that prolonged exposure to mucus preferentially reduced the transport of PPZ‐decorated particles. PPZ functionalization improves colloidal stability and reduces gross mucin‐induced aggregation (favorable for initial penetration), but the same multivalent, cationic surface may promote progressive, localized interactions and retention within the mucus/epithelium matrix during prolonged exposure. Unmodified LPSNP, while more susceptible to mucin‐induced size increase in bulk, may experience weaker long‐term binding and less retention, resulting in higher apparent permeability at late time points. However, this trend reflects the time‐dependent diffusion and depletion of nanoparticles from the apical compartment rather than an abrupt loss of enhancer function or an instantaneous on/off state of the mucus barrier. Upon exposure, LPSNP‐PPZ is expected to induce time‐dependent changes in both the mucus and epithelial compartments, such as rearrangement or partial loosening of the mucin network and transient modulation of epithelial junctions and uptake pathways. These alterations do not vanish immediately when the measured nanoparticle penetration rate starts to decline; instead, they persist over a finite ‘enhancement window’ during which the barrier remains more permissive than under control conditions.

Taken together, the mucin co‐incubation and mucus‐permeability data indicate that PPZ functionalization mainly modulates how LPSNP interacts with the mucus mesh rather than their intrinsic colloidal robustness. In excess soluble mucin, LPSNP‐PPZ remain near their initial size while unmodified LPSNP undergo mucin‐driven aggregation, and at early time points in the mucus transport assay, LPSNP‐PPZ_H_ show slightly enhanced flux, consistent with reduced large‐scale clustering. However, during prolonged exposure, the same multivalent piperazine surface likely promotes progressive, localized binding and retention within the mucus/epithelium matrix, leading to lower net permeability than the weaklier interacting LPSNP at 3 h. In line with this interpretation, when the same formulations are placed in simulated intestinal fluid containing digestive enzymes but no structured mucus network, all particles—irrespective of PPZ content—display only modest, self‐limited increases in hydrodynamic diameter that stabilize below 200 nm over 24 h, with minimal PDI changes and no evidence of large aggregates (Figure S9). The PDI values showed only a slight elevation at 24 h but stayed within a range typically considered acceptable for nanosuspensions, indicating limited broadening of the size distribution rather than major instability. Thus, under enzyme‐rich but mucus‐poor intestinal conditions, PPZ‐modified LPSNP behaves as colloidally stable nanosuspensions, and the differences observed in mucus transport can be attributed primarily to differential muco‐interactions rather than to any intrinsic instability of the PPZ coatings.

### 2.4 In vitro Cytotoxicity

Next, we assessed the cytotoxicity of LPSNP, PPZ‐functionalized LPSNP, and PPZ against Caco‐2 cells and HT29‐MTX cells. Caco‐2 cells, derived from colorectal adenocarcinoma, represent a well‐established cell line widely employed for intestinal epithelial absorption and oral drug delivery research [49]. HT29‐MTX is a subclone of the HT29 cells, human colon adenocarcinoma cells that have been induced by methotrexate. These cells closely resemble the intestinal epithelial morphology in vivo and also secrete mucus [50]. Various concentrations of LPSNP, PPZ functionalized LPSNP, and PPZ were incubated with Caco‐2 cells and HT29‐MTX cells. The cell viability is shown in Figure S10A for Caco‐2 cells and Figure S10B for HT29‐MTX cells. The results demonstrated that LPSNP and PPZ functionalized LPSNP did not induce any cytotoxicity in either cell line (0.1–2 mg/mL). While the nanoparticles demonstrated no toxicity to the cells, PPZ solution induced significant cytotoxicity, causing a 50% reduction in Caco‐2 cell viability and 75% reduction in HT29‐MTX cell viability at a 5 mm (0.081 mg/mL) concentration. This result agreed with the literature, which showed 6 mM PPZ induced ex vivo tissue damage [17]. Additional viability assays were performed after 3 and 7 days of continuous culture in the presence of the formulations (Figures S11 and S12). No significant cytotoxicity was detected for any nanoparticle group at day 3, with cell viability remaining comparable to untreated controls. At day 7, LPSNP‐PPZ_L_ induced only a slight reduction in HT29‐MTX cell viability, which nevertheless remained above 80%, whereas the other formulations did not show a significant loss of viability. These findings support that the nanoparticles do not cause pronounced long‐term cytotoxicity. Subsequent confocal microscopy analysis revealed the well‐defined staining of actin filaments around Caco‐2 cells using phalloidin (Figure S13) [51]. PPZ was not toxic to Caco‐2 cells at 1 mM, however 5 mM and 10 mM concentrations caused significant reductions in cell number and cell actin. Neither pristine silica nanoparticles (LPSNP) nor PPZ‐functionalized LPSNP (2 mg/mL) exhibited any significant reduction in cell number or actin loss in either Caco‐2 or HT29‐MTX cells (Figure S14). Quantification of fluorescent signal also indicated a significant decrease in the phalloidin/DAPI ratio, in both Caco‐2 and HT29‐MTX cell lines (Figure S15). These findings align with the MTS results, indicating that PPZ‐functionalized LPSNP are non‐toxic to both cell lines and could potentially serve as a safe permeation enhancer.

### 2.5 Effect of LPSNP‐PPZ Particles on In vitro Monolayers

**FIGURE 4:**
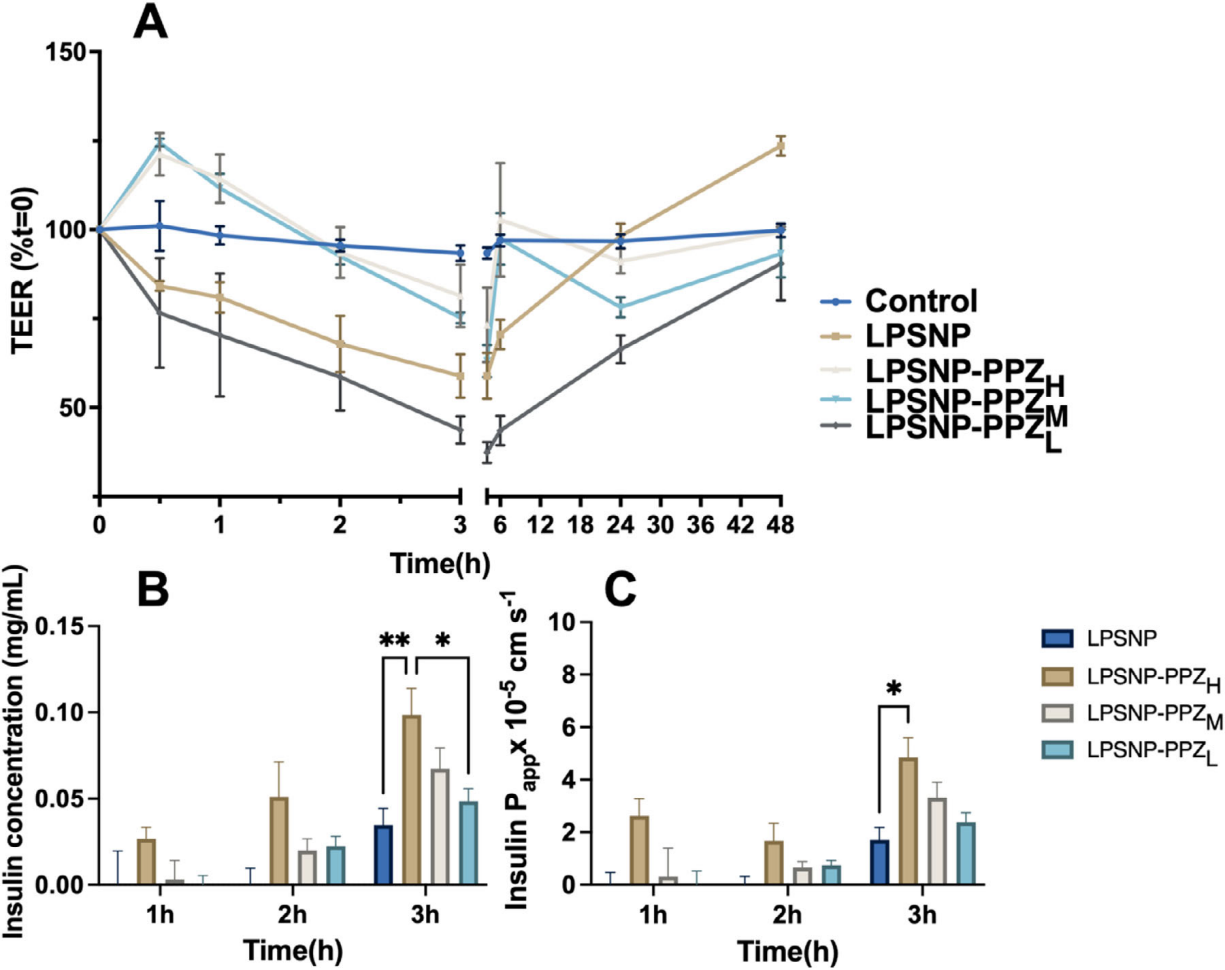
TEER value change of different silica nanoparticles on in vitro Caco‐2 monolayers (A). The concentration of insulin in the basolateral chamber and its related Papp value (B) and (C), respectively. The data was subtracted from the insulin permeation amount in the control group (Caco‐2 monolayer with insulin alone). *n* = 3, mean + SEM, ^*^ *p* < 0.05, ^**^ *p* < 0.01, ^***^ *p* < 0.001.

Caco‐2 cell monolayer has been extensively used in vitro in the screening of drug permeability and absorption of orally administered drugs [52]. When cultured on Transwell inserts, Caco‐2 cells spontaneously differentiate into polarized monolayers, forming a physical and biochemical barrier that closely resembles the intestinal epithelium in vivo [53]. To evaluate the integrity of the cellular barriers, various concentrations of silica nanoparticles were applied to the apical side of the Caco‐2 cell monolayer, and trans‐epithelial electrical resistance (TEER) measurements were conducted [54]. Treatment with nanoparticles (Figure 4A), LPSNP, and LPSNP‐PPZ_L_ exhibited a significant decline in TEER, but there were no significant differences between the different nanoparticle types. After 3 h of treatment, the nanoparticles were removed, and the TEER values fully recovered within 48 h. The transit TEER value change indicated LPSNP‐PPZ as a potential reversible, non‐toxic permeation enhancer for large molecules. Furthermore, we evaluated the ability of the nanoparticles to effectively enhance insulin permeation across the epithelial barrier by co‐treating the epithelial cell monolayers with insulin and nanoparticles. LPSNP‐PPZ_M_ and LPSNP‐PPZ_H_ induced a modest TEER increase at first, followed by a reversible decrease at later time points. This biphasic behavior likely reflects an initial adaptive tightening of the tight junction complex and/or changes in ion transport in response to nanoparticle exposure, as previously observed for other barrier models exposed to nanomaterials, before subsequent signalling events promote transient junctional opening and enhanced paracellular permeability [55, 56]. With the treatment of nanoparticles on the Caco‐2 model (Figure 4B), insulin permeability was low at 1 h. At 3 h, LPSNP‐PPZ_H_ induced the highest insulin permeation. The apparent permeability coefficients (Papp) indicated at 3 h, insulin is highly permeable with LPSNP‐PPZ_H_ (Papp = 4.5 × 10^−5^ cm/s) and significantly higher than the pristine silica nanoparticles (Papp = 2.6 × 10^−5^ cm/s) (Figure 4C). The data suggested that the insulin has a high Caco‐2 permeability when co‐administered with nanoparticles [57]. In contrast, only 10 mm PPZ was able to significantly reduce the TEER value (Figure S16A), a concentration that also showed high cytotoxicity.

Despite its widespread use in in vitro intestinal permeation studies, the Caco‐2 monolayer exhibits minimal expression of gel‐forming mucin genes and is classified as a non/low‐mucus producing cell line [58]. Therefore, to further investigate the permeability of insulin with silica nanoparticles as a permeation enhancer through an intact secreted mucus layer, the Caco‐2/HT29‐MTX co‐culture model was utilised [59, 60]. Co‐treatment with silica nanoparticles and insulin resulted in a decrease in TEER values (Figure S17A). The PPZ functionalized LPSNP exhibited significantly lower TEER values compared to the pristine LPSNP, indicating enhanced permeation ability. This was further supported by the higher permeation of insulin concentration (Figure S17B) and higher Papp value (Figure S17C). The in vitro results demonstrated the potential of PPZ functionalized to LPSNPs to enhance the insulin permeability. However, the impact of varying PPZ grafting densities on LPSNP remained unclear. In both Caco‐2 and Caco‐2/HT29‐MTX monolayers, LPSNP‐PPZ_L_ showed a slightly greater ability to reduce TEER values, while LPSNP‐PPZ_H_ facilitated higher insulin permeation across the monolayer. This finding suggests that the hydrophobicity of LPSNP‐PPZ_H_ may influence their interactions with the monolayers; moreover, the high surface density of piperazine groups on these nanoparticles could modulate their interactions with cellular membranes and associated intracellular pathways [61, 62]. The role of particle size and surface morphology in our system aligns with previous investigations on mesoporous silica nanoparticles in intestinal models [25, 63, 64, 65]. Our dendritic LPSNPs have an overall diameter of approximately 100 nm with radially oriented mesopores of 7–8 nm, a size regime that is compatible with diffusion through the mucus mesh while still allowing intimate contact with the epithelial surface. Within this design window, nanoparticles are small enough to avoid extensive steric hindrance in mucus but large enough to effectively engage the cell membrane and junctional complexes. Moreover, the dendritic architecture substantially increases the accessible surface area and generates a rough, corrugated interface that presents a high density of piperazine groups toward the biological milieu. This combination of 100 nm size and nanoscale surface roughness is consistent with reports that rough or dendritic mesoporous silica particles interact more strongly with epithelial cells and can more efficiently induce transient tight junction opening than smooth, low–surface‐area counterparts [4]. The reversible reductions in TEER and enhanced paracellular permeability observed for LPSNP‐PPZ_H_ arise from the synergistic interplay between their nanoscale (≈100 nm) size, which enables efficient mucus penetration and intimate epithelial contact, and their uniquely engineered rough dendritic morphology, which increases the surface exposure of piperazine groups. This combination yields a distinct capacity to transiently modulate tight junctions compared with smooth nanoparticles, representing a novel structure–function design that integrates surface roughness and high‐density ionizable groups to achieve controlled epithelial permeation. In both Caco‐2 and Caco‐2/HT29‐MTX layers, exposure to LPSNP‐PPZ led to a transient disruption of the continuous ZO‐1 belt at intercellular junctions, with a more discontinuous, patchy pattern that paralleled the reduction in TEER (Figures S18 and S19). These findings provide direct mechanistic evidence that LPSNP‐PPZ regulates tight junction architecture to facilitate paracellular transport. Due to the limitations of in vitro models, further investigation was warranted to evaluate the permeation enhancement efficacy of LPSNP‐PPZ_H_ and LPSNP‐PPZ_L_ in vivo*_._*

### 2.6 LPSNP‐PPZ Nanoparticles Facilitate Enhance Oral Absorption of Macromolecule in Mice

**FIGURE 5:**
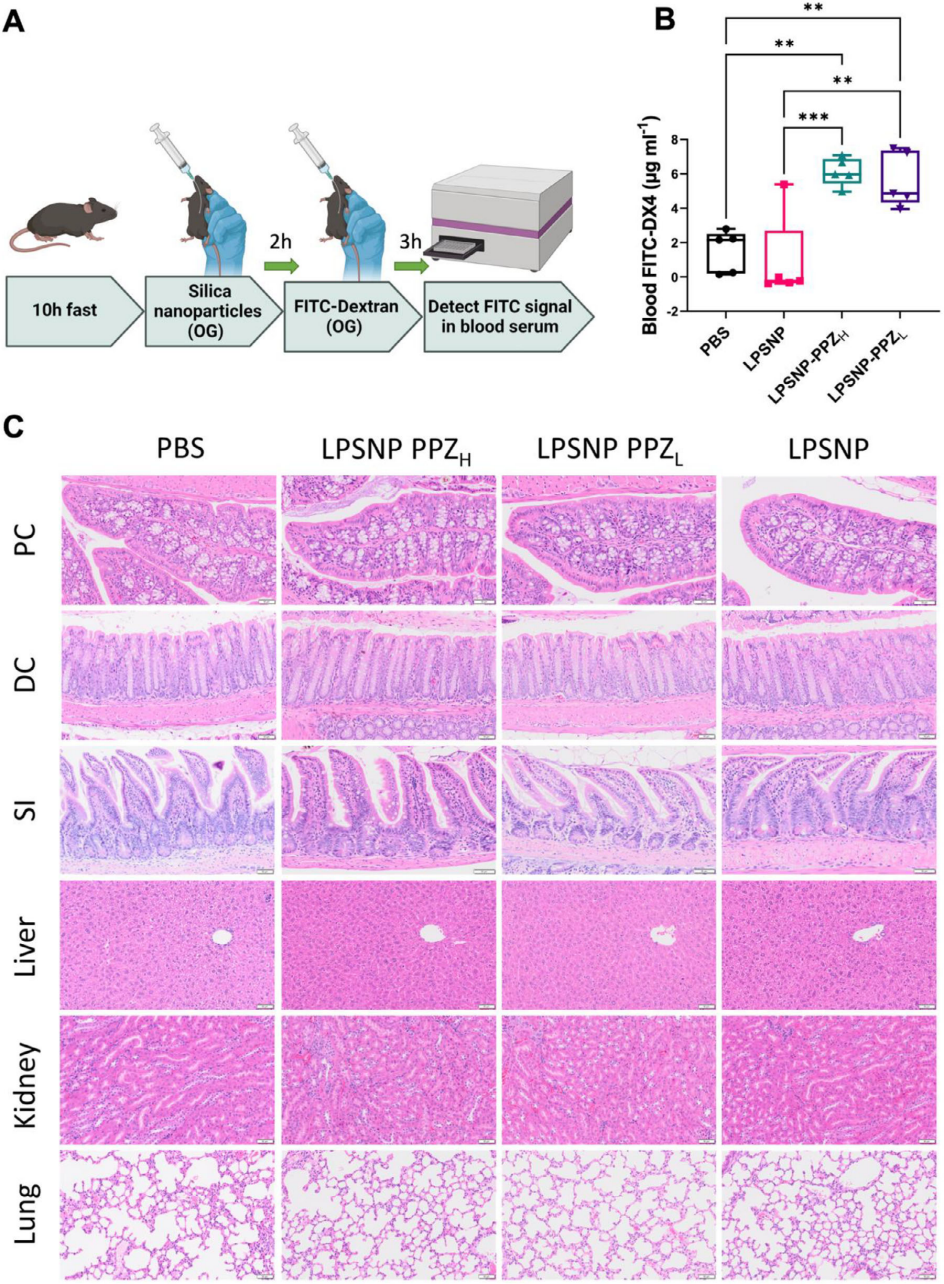
(A) Scheme of in vivo experiment, OG: oral gavage. Created with BioRender.com (B) Oral administration of pristine LPSNP and PPZ functionalized LPSNP at 100 mg/kg (*n* = 5) improves oral absorption of FITC‐DX4 in the serum. ^**^ *p* < 0.01, ^***^ *p* < 0.001 (C) Representative H&E staining of the PC: proximal colon, DC: distal colon, SI: small intestine, liver, kidney, and lung showed no overt signs of acute toxicity in mice (scale bar 50 µm).

We then investigated the in vivo permeation enhancement effect of pristine LPSNP and PPZ functionalized LPSNP using FITC‐Dextran 4 kDa (FITC‐DX4) as a reference macromolecule. FITC‐DX4 was employed as an indicator due to its comparable molecular weight to insulin (5.8 kDa) and its ability to resist the acidic and enzymatic conditions of the stomach and small intestine [9, 25]. Following a 10 h fast, mice were orally gavaged with various types of silica nanoparticles (100 mg/kg). The oral dose of 100 mg/kg nanoparticles was selected based on previously published studies using related silica–based formulations [25], which demonstrated acceptable safety and tolerability at this level, including under repeated dosing [63]. Two hours later FITC‐DX4 was administered via oral gavage, and the absorption of FITC‐DX4 was evaluated by FITC level in the serum after 3 h (Figure 5A). LPSNP‐PPZ_H_ and LPSNP‐PPZ_L_ significantly increased FITC‐DX4 absorption compared to LPSNP and the PBS control (Figure 5B). While LPSNP‐PPZ_H_ exhibited a slightly higher average absorption than LPSNP‐PPZ_L_, the difference was not statistically significant. Histological analyses of the colon (proximal and distal), small intestine, liver, kidney, and lung did not show any acute toxicity such as cellular damage or immune cell infiltration (Figure 5C). Overall, the preliminary in vivo data demonstrate the ability of LPSNP‐PPZ to enhance the absorption of the macromolecule FITC‐DX4 without any acute toxicity after a single dose, corroborating our hypothesis [9].

As mentioned before, insulin injection is currently one of the most effective ways to control the blood glucose level in Type 1 diabetes patients, but currently, there are still no commercially available oral insulin formulations. Having confirmed the successful absorption of oral FITC‐DX4 via the gastrointestinal tract (Figure 5B), we next examined the oral delivery of insulin with PPZ‐functionalized silica nanoparticles in a high‐fat diet mouse model. To protect insulin from the highly acidic environment of the stomach, it was encapsulated with a size M mice capsule along with a protease inhibitor and subsequently coated with Eudragit L100‐55 to achieve pH‐responsive release [66, 67]. As shown in Figure S20, the coated size M mice capsule remained intact without any drug released after being stirred in a pH 1.9 buffer for 30 min (floating on the top of the solution), while the uncoated capsule dissolved and released insulin. This observation demonstrates the effectiveness of Eudragit L100‐55 in protecting the drug and excipients from the acidic environment in the stomach [68, 69]. To confirm insulin release kinetics from the Eudragit‐coated capsules, an in vitro release study was conducted under pH conditions simulating gastrointestinal transit (Figure S21). Insulin remained largely retained under simulated gastric conditions (pH 1.9) for 1 h, consistent with the typical mouse gastric half‐emptying time of 1–1.5 h [70], and exhibited a burst release within 30 min upon shifting to intestinal pH (pH 7.4), reaching complete (100%) release by 1 h. These profiles align with the pH‐responsive dissolution properties of the Eudragit coating and indicate that insulin becomes bioavailable predominantly in the intestine.

The majority of studies on oral insulin delivery utilised either chemically induced type II diabetes models or healthy mice to simply measure the bioavailability of insulin. However, models where insulin resistance is coupled with hyperglycaemia had not been tested. Therefore, to test our delivery system and its ability to reduce hyperglycaemia effectively, we utilised the high‐fat diet (HFD) mouse model [71, 72, 73]. The mice were fed with HFD (containing 46% of available energy as saturated fat, 20% protein, and 4.80% crude fibre) for 12 weeks to induce pre‐diabetes. As shown in Figure S22, compared with control (normal chow diet, NCD) mice, the HFD mice indicated higher body weight in 12 weeks. Moreover, the glucose tolerance test (GTT) showed that after a 2 h intraperitoneal (IP) injection of glucose (0.75 U/kg), the blood glucose levels in normal chow diet mice returned to baseline, whereas in HFD mice, glucose levels remained elevated at 13.32 mmol/L, higher than fasting plasma glucose (Figure S22B). The IP insulin tolerance test (IPITT) showed that both NCD and HFD mice experienced a significant blood glucose reduction within 30 min. However, HFD mice exhibited a less pronounced decline, demonstrating insulin resistance (Figure S22C) but still showing a response to insulin.

**FIGURE 6:**
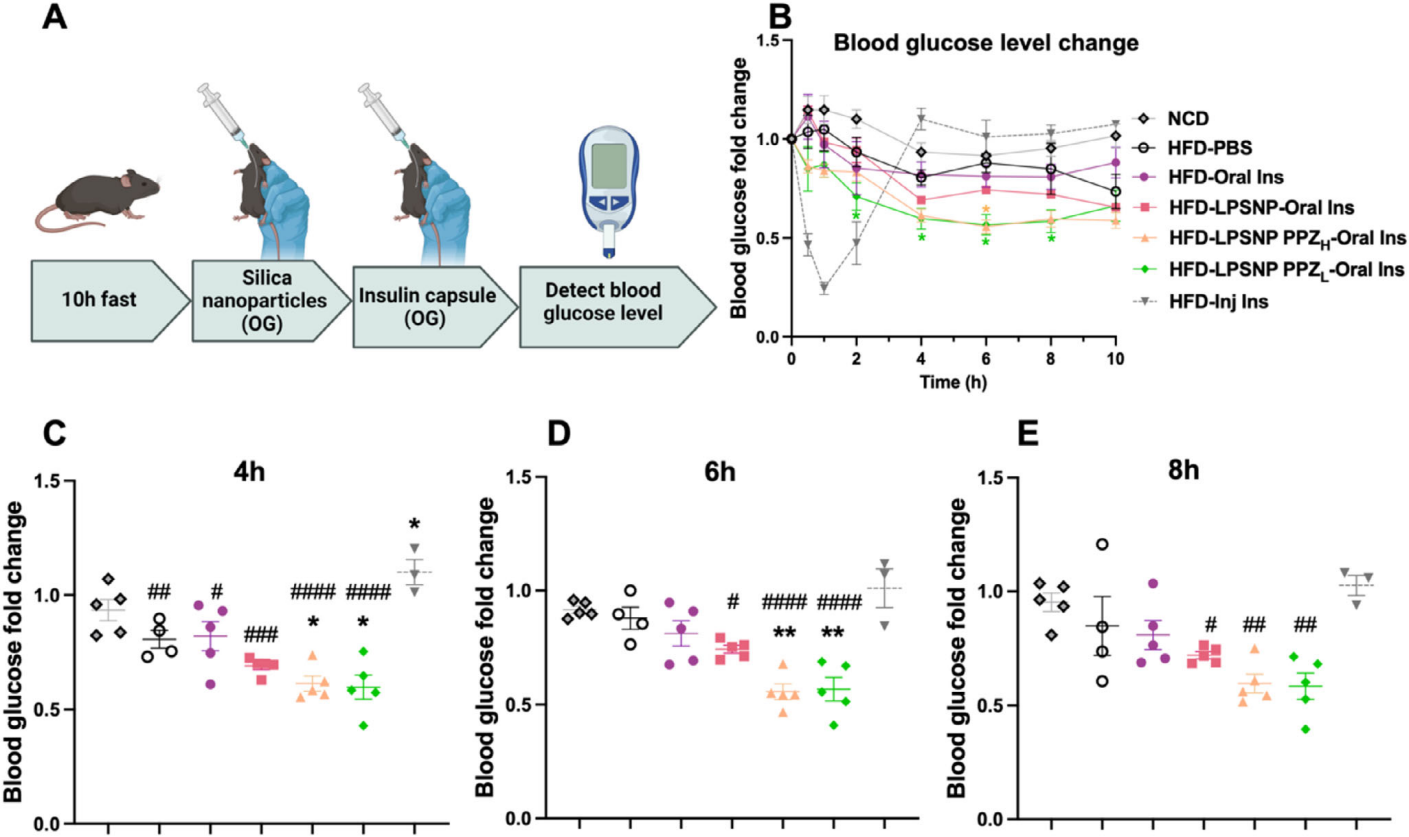
(A) Scheme of in vivo experiment with high‐fat diet (HFD) fed mice treated with silica nanoparticles and insulin capsule, OG: oral gavage. (B) Blood glucose level fold change after oral gavage of different silica nanoparticles and insulin capsules. *n* = 5 except HFD‐PBS (*n* = 4) and HFD‐Inj Ins (*n* = 3), Mean ± SD. (C–E) Blood glucose level fold change comparison after 4, 6, and 8 h oral gavage of an insulin capsule. Administration of LPSNP‐PPZ_H_/ LPSNP‐PPZ_L_ with oral insulin capsule showed significant blood glucose level reduction and sustained blood glucose level control. *Represents the significant difference compared with HFD‐Oral Ins group, ^*^ *p* < 0.05, ^**^ *p* < 0.01; # Represents the significant difference compared with HFD‐Inj Ins group, ^#^ *p* < 0.05, ^##^ *p* < 0.01, ^###^ *p* < 0.001, ^####^ *p* < 0.0001.

**FIGURE 7:**
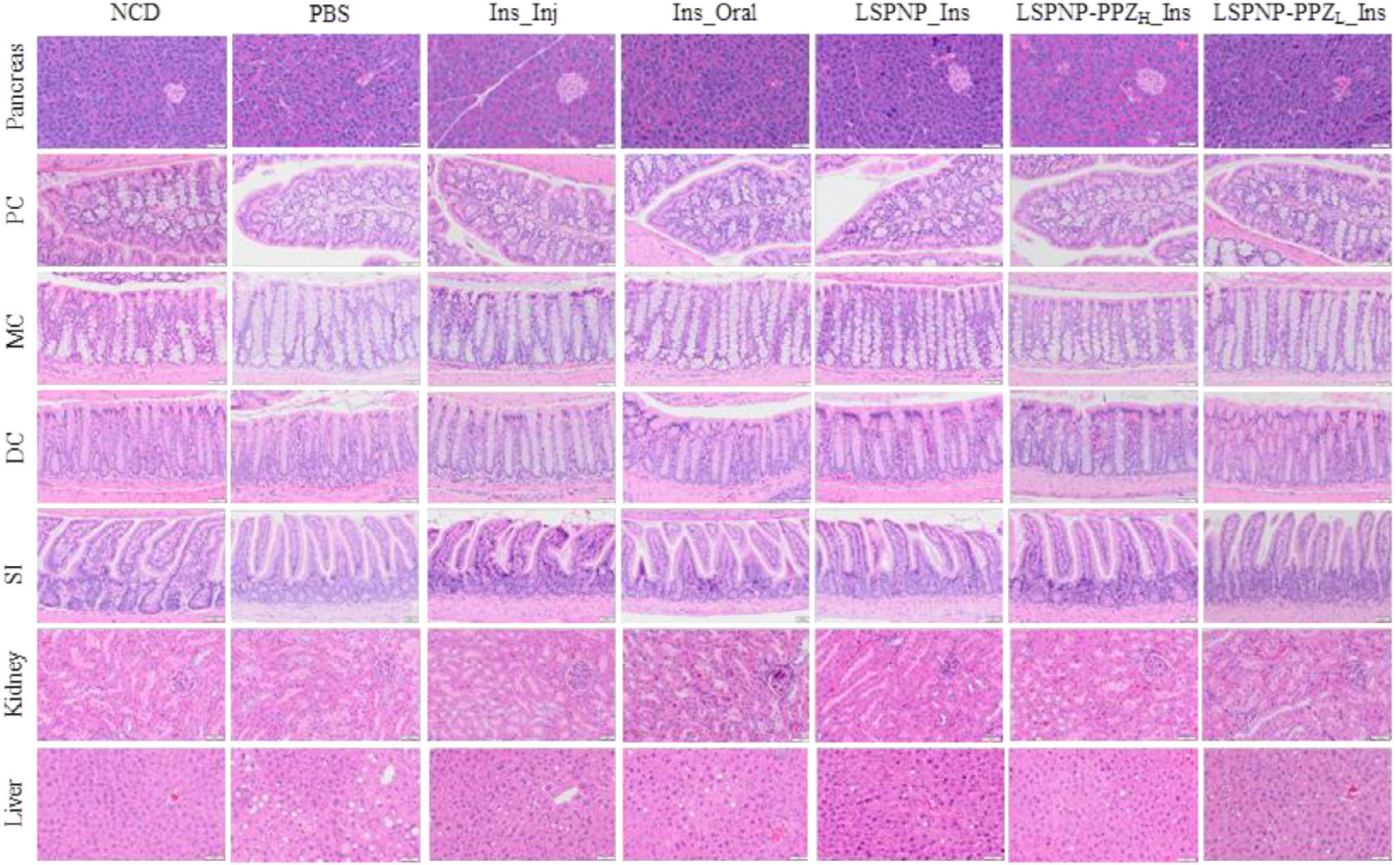
Representative H&E staining of the pancreas, proximal colon (PC), middle colon (MC), distal colon (DC), small intestinal (SI), liver, and kidney showed no direct overt signs of systemic toxicity to acute toxicity in mice (scale bar 50 µm). NCD: normal chow diet. Other groups were treated under high fat diet.

To evaluate the effectiveness of PPZ functionalized silica nanoparticles in enhancing oral insulin delivery, we used HFD mice to mimic pre‐diabetes. Prior to the experiment, the mice were subjected to a 10 h fast to guarantee an empty stomach for optimal nanoparticle absorption and to minimize the impact of recent food intake on absorption and metabolism, ensuring more comparable data. Two hours following oral gavage with nanoparticles (100 mg/kg), insulin capsules (675 U/kg) were administered orally. This timing aligns with evidence indicating that silica nanoparticles induce the highest permeability of the mouse intestine within a 2 h window [25] (schematic in Figure 6A). Although LPSNP possesses a porous architecture, in the present study, the pores were not exploited for insulin loading. Instead, the porous structure is expected to influence the effective surface area, local hydration, and accessibility of the piperazine‐functionalized shell, thereby enhancing the drug across the epithelial barrier with low toxicity. Blood glucose levels were monitored at 30 min, 1, 2, 4, 6, 8, and 10 h following insulin administration (Figure 6B). The results revealed that PPZ functionalized LPSNPs with both high (LPSNP‐PPZ_H_) and low (LPSNP‐PPZ_L_) grafting densities effectively reduced blood glucose levels for up to 8–10 h compared to LPSNP alone. It is noteworthy that the insulin injection group exhibited a rapid decline in blood glucose levels, returning to normal after 4 h, as expected. At 4 and 6 h, the LPSNP‐PPZ_H_ and LPSNP‐PPZ_L_ with oral insulin groups demonstrated significantly lower blood glucose levels compared to the oral insulin‐only group at the same dose (Figure 6C, D, data are presented as fold change of starting blood glucose concentrations); this effect persisted until 10 h. Importantly, H&E staining from different organs: pancreas, proximal colon (PC), middle colon (MC), distal colon (DC), small intestinal (SI), liver, and kidney confirmed that no acute toxicity or apparent alterations in immune cell infiltrates were observed with any of the treatments (Figure 7). These findings suggest that PPZ functionalized LPSNPs can enhance oral insulin absorption and prolong its therapeutic effects.

As LPSNP‐PPZ is used as a permeation enhancer for oral macromolecular drug delivery, its in vivo residence time, degradability, and long‐term safety are critical considerations. Extensive prior work on silica nanoparticles provides a strong framework to contextualize the biological fate of this material [63]. In previous studies, orally administered amorphous silica nanoparticles typically exhibit a short residence time in the gastrointestinal tract, with the majority of particles eliminated via faecal excretion within hours to days [74, 75]. Particles that transiently interact with or are internalized by intestinal epithelial cells or immune cells show minimal systemic exposure, with biodistribution largely restricted to clearance organs such as the liver and spleen [76]. Importantly, oral delivery results in substantially lower systemic retention compared with intravenous routes [77]. Nevertheless, silica nanoparticles are biodegradable under physiological conditions. Hydrolytic cleavage of Si–O–Si bonds leads to gradual dissolution into orthosilicic acid (Si(OH)_4_), a naturally occurring, water‐soluble silicon species that is readily cleared via renal excretion [78]. Degradation kinetics depend on particle size, porosity, degree of condensation, and surface chemistry. While organic surface functionalization, such as piperazine grafting in LPSNP‐PPZ, may moderately slow degradation by partially shielding the silica surface, it does not prevent eventual breakdown into biocompatible silicon species. More importantly, it is emphasized that amorphous silica does not share the toxicity profile of crystalline silica, which is associated with silicosis upon inhalation. Oral exposure to amorphous and mesoporous silica nanoparticles has demonstrated excellent tolerability in multiple animal studies and human clinical trials, even at doses far exceeding those required for drug delivery or permeation enhancement [79, 80]. Notably, oral clinical studies using mesoporous and hybrid silica nanoparticles reported no significant adverse effects, supporting the conclusion that these materials do not accumulate to harmful levels in the body [26]. Silica nanoparticles are generally recognized as safe (GRAS), with decades of use as pharmaceutical excipients and food additives (e.g., E551), and several silica‐based oral formulations have advanced into Phase I and II clinical trials without safety concerns. As a permeation enhancer, LPSNP‐PPZ functions as a transient permeation enhancer rather than a long‐circulating carrier, further reducing safety risks. Taken together, existing evidence indicates that LPSNP‐PPZ is likely to exhibit transient residence within the gastrointestinal tract, undergo gradual biodegradation into biocompatible silicon species, and be cleared from the body. While available evidence supports the favorable short‐term safety and clearance of silica‐based nanoparticles, potential long‐term safety considerations for LPSNP‐PPZ include cumulative exposure effects following repeated oral dosing, delayed biodegradation associated with surface functionalization, and prolonged interactions with intestinal epithelial cells and mucosal immune populations. Future work will include repeated‐dose oral toxicity studies over extended durations, comprehensive histopathological analysis of gastrointestinal tissues and major clearance organs, biodistribution and degradation profiling using silicon quantification methods, and detailed immunological assessments encompassing cytokine production, immune cell phenotyping, and evaluation of mucosal immune activation. In addition, functional assays to assess epithelial barrier integrity, mucus layer recovery, and microbiota composition following prolonged exposure will be conducted to fully define the long‐term safety and translational potential of LPSNP‐PPZ.

## 3 Conclusions

In this study, we explored the potential of 1‐phenylpiperazine (PPZ) functionalized large pore silica nanoparticles (LPSNP‐PPZ) as a novel permeation enhancer for an oral macromolecular drug delivery system. Our synthesis of PPZ silane, characterised by high reproducibility and by‐product‐free formation, laid the foundation for the development of LPSNP‐PPZ. Our investigation, encompassing both in vitro and in vivo models, revealed the remarkable ability of LPSNP‐PPZ to enhance intestinal permeability and promote macromolecule absorption via the paracellular route. Transwell models employing Caco‐2 and Caco‐2/HT29‐MTX monolayers demonstrated that LPSNP‐PPZ could temperately decrease the TEER value, thus leading to transit‐enhancing tight junction opening, paving the way for enhanced macromolecule transport. In vivo studies further corroborated the efficacy of LPSNP‐PPZ. Compared to pristine LPSNP, LPSNP‐PPZ facilitated significantly higher absorption of FITC‐Dex (4 kDa) across the intestinal barrier. Although at a higher dose compared to IP insulin, LPSNP‐PPZ enhanced the absorption of oral insulin from capsules in high‐fat diet mice, demonstrating its potential as a therapeutic agent.

This study underscores the significant potential of LPSNP‐PPZ as an innovative and promising oral delivery platform for macromolecules. This novel system presents a viable alternative to traditional approaches that involve the use of chemical permeation enhancers, which have been associated with challenges such as potential irreversible damage to epithelial cells. LPSNP‐PPZ demonstrates the capacity to address these challenges by providing an effective means of transporting macromolecules without significantly compromising the integrity of the epithelial barrier. This breakthrough not only highlights the potential of LPSNP‐PPZ for enhanced drug delivery but also suggests a safer and more efficient approach, positioning it as a noteworthy advancement in the field of oral drug delivery systems. Despite being tested on the intestinal barrier, we foresee that these findings could be relevant to other barriers, such as the blood–brain barrier and lung epithelial barriers.

## Conflicts of Interest

The authors declare no conflicts of interest.

## Supporting information




**Supporting File 1**: advs74624‐sup‐0001‐SuppMat.docx.

## Data Availability

The data that support the findings of this study are available from the corresponding author upon reasonable request.
