# Supplementary material for: Piperazine‐Functionalized Nanoparticles Enable Oral Insulin Delivery in Obese Mice
Source: Adv Sci (Weinh). 2026 Mar 7;13(27):e20918. doi: 10.1002/advs.202520918 (PMC13170209; doi:10.1002/advs.202520918)
Supplement: Supplementary file 1 — Supporting File 1: advs74624‐sup‐0001‐SuppMat.docx. [file ADVS-13-e20918-s001.docx]

*Supporting Information for*

**Piperazine-Functionalized Nanoparticles Enable Oral Insulin Delivery in Obese Mice**

Yuxue Cao^a,b^, Xiaofan Jiang^a^, Md Moniruzzaman^c^, Alexandra Muller^d^, Taskeen Iqbal Janjua^a^, Nisha Tyagi^a^, Zhi Qu^a^, Kuan Yau Wong^d^, Yuran Feng^a^, Aayushi Ghodasara^a^, Ekaterina Strounina^e^, Benjamin P. Ross*^a^, Tushar Kumeria*^f,g^, Sumaira Z. Hasnain*^d,h^, Amirali Popat*^a,i^

[a] Dr. Y. Cao, X. Jiang, Dr. T. Janjua, N. Tyagi, Dr. Z. Qu, Y. Feng, A. Ghodasara, Dr. B. P. Ross, Prof. A. Popat
School of Pharmacy and Pharmaceutical Sciences, The University of Queensland , Brisbane, QLD 4102, Australia
E-mail: [b.ross1@uq.edu.au](mailto:b.ross1@uq.edu.au), [a.popat@uq.edu.au](mailto:a.popat@uq.edu.au)

[b] Dr. Y. Cao
Drug Delivery, Disposition and Dynamics, Monash Institute of Pharmaceutical Sciences, Monash University, Parkville, VIC 3052, Australia

[c] Dr. M. Moniruzzaman

Faculty of Medicine, The University of Queensland, 37 Kent Street, Woolloongabba, QLD 4102, Australia

[d] A. Muller, Dr. K. Y. Wong, Prof. S. Z. Hasnain

Immunopathology Group, Mater Research Institute, The University of Queensland, Translational Research Institute, Brisbane, QLD, 4102, Australia.

E-mail: [sumaira.hasnain@mater.uq.edu.au](mailto:sumaira.hasnain@mater.uq.edu.au)

[e] Dr. E. Strounina

Centre for Advanced Imaging, The University of Queensland QLD 4072, Australia

[f] A/Prof. T. Kumeria

School of Materials Science and Engineering, The University of New South Wales, Sydney, NSW, 2052, Australia.

E-mail: [t.kumeria@unsw.edu.au](mailto:t.kumeria@unsw.edu.au)

[g] A/Prof. T. Kumeria

Australian Centre for Nanomedicine, The University of New South Wales, Sydney, NSW 2052, Australia

E-mail: [t.kumeria@unsw.edu.au](mailto:t.kumeria@unsw.edu.au)

[h] Prof. S. Z. Hasnain

Australian Infectious Diseases Research Centre, The University of Queensland, Brisbane, QLD, 4102, Australia

[i] Prof. A. Popat

ADepartment of Functional Materials and Catalysis, Faculty of Chemistry, University of Vienna, Währinger Straße 42, 1090 Vienna, Austria

# Materials and method

## Materials

(3-Iodopropyl)trimethoxysilane, 1-phenylpiperazine, triethylamine, cetrimonium chloride, triethanolamine, tetraethoxysilane, sodium pyruvate solution, toluene, sodium butyrate, bovine serum albumin (BSA), mucin from porcine stomach, metoclopramide hydrochloride, aprotinin and FITC-carboxymethyl dextran 4 kDa were purchased from Sigma-Aldrich, Australia. For syntheses that require anhydrous conditions, solvents were dried over molecular sieves (4 Å). Chloroform-d (CDCl_3_) was purchased from Cambridge Isotope Laboratories. Dulbecco's Modified Eagle Medium (DMEM), Minimal Essential Medium (MEM), glutamine, penicillin/streptomycin (P/S), and 0.25% trypsin–EDTA were purchased from Life Technologies. Hanks’ Balanced Salt Solution (HBSS) and HEPES buffer (4-(2-hydroxyethyl)-1-piperazineethanesulfonic acid) were purchased from Gibco. MTS Assay Kit (Cell Proliferation) was purchased from Abcam. For staining solutions, Alexa Fluor™ 488 Phalloidin, DAPI (4′,6-diamidino-2-phenylindole), Alexa Fluor 594 conjugated anti-zonula occludens-1 (ZO-1) antibodies, and Alexa Fluor 488 conjugated anti-claudin 5 antibodies were purchased from Thermofisher, Australia. Rat tail collagen I was purchased from Thermofisher, Australia. Mouse-sized (M) capsules and dosing kits were brought from Torpac company and Eudragit L100-55 enteric coating polymers were kindly received as samples from Evonik. Blood glucose strips and a blood glucose monitor (Senso card Plus) for the animal experiment were obtained from Point of Care Diagnostics, New Zealand.

## Synthesis of 1-phenyl-4-(3-(trimethoxysilyl)propyl)piperazine

The procedure for synthesis of 1-phenyl-4-(3-(trimethoxysilyl)propyl)piperazine was adapted from the methods of Kiso (2008). (3-Iodopropyl)trimethoxysilane (3.9 mL, 20 mmol) was slowly added to a mixture of 1-phenylpiperazine (3.1 mL, 20 mmol) and triethylamine (2.8 mL, 20 mmol) under nitrogen while stirring. The resultant yellow slurry was stirred at room temperature for 30 min until the yellow colour disappeared, then the white slurry was kept at -20 °C overnight. The solid precipitate was collected by filtration and washed with hexane. The combined filtrates were concentrated under reduced pressure to a pale-yellow oil, which was purified by vacuum (Kugelrohr) distillation to afford a colourless oil (1.44 g, 22.4%). The product was analyzed by NMR spectroscopy using a Bruker AV300 spectrometer and TopSpin 4.1.1 pl 7 for processing spectra, with the solvent residual peak (CDCl_3_, ^1^H NMR δ 7.26 ppm or ^13^C NMR δ 77.16 ppm) as the internal reference: ^1^H NMR [300 MHz, CDCl_3_] δ 7.30-7.21 (m, 2H), 6.96-6.82 (m, 3H), 3.57 (s, 9H), 3.43-3.15 (m, 4H), 2.90-2.64 (m, 4H), 2.64-2.42 (m, 2H), 1.86-1.62 (m, 2H), 0.72-0.58 (m, 2H) ppm. ^13^C NMR [75 MHz, CDCl_3_] δ 151.02, 129.29, 120.27, 116.46, 61.01, 52.92, 50.72, 48.67, 19.41, 6.82 ppm. The product was stored at -20 °C under inert nitrogen atmosphere until further use.

## Synthesis of large pore silica nanoparticles

Large pore silica nanoparticles (LPSNP) were synthesized using our reported method with modification [1]. Briefly, 20 mL cetrimonium chloride (CTAC), 0.9 g triethanolamine (TEA), and 90 mL Milli-Q water were mixed and stirred at 375 rpm and 60 °C for 1 h. After that, pre-mixed 80 mL cyclohexane and 20 mL of TEOS were added and kept stirring for 12 h. The product was collected by centrifugation with high-speed ultra-centrifugation (Jouan Centrifuge KR22i in the rotor AK50-22) at 24,700$g$ for 10 min, washed three times with ethanol, and dried in an oven at 60°C. To remove the surfactant, the nanoparticles were calcined in a muffle furnace (Thermo Scientific, Australia) by increasing temperature from ambient up to 550 °C at a rate of 10 °C/min and maintaining 550 °C for 5 h.

## Silica nanoparticle functionalization

1-Phenylpiperazine was grafted onto the LPSNP using the following method to achieve high grafting density. LPSNP (100 mg) were dispersed in 15 mL dry toluene and then refluxed for 24 h under an atmosphere of nitrogen. Then, 1-phenyl-4-(3-(trimethoxysilyl)propyl)piperazine (100 µL) was added followed by further refluxing for 24 h. The final product (LPSNP-PPZ_H_) was washed with 70% ethanol at least 3 times to remove residuals and collected by centrifugation at 24,700 $\times$ g for 10 min, and finally vacuum dried at room temperature for 24 h. For other grafting densities: LPSNP-PPZ_M_ (medium grafting density) and LPSNP-PPZ_L_ (low grafting density), 50 µL or 10 µL of 1-phenyl-4-(3-(trimethoxysilyl)propyl)piperazine, respectively, was added into 100 mg of the LPSNP, while other steps remained the same.

## Surface hydrophobic/hydrophilic properties

An ethanolic suspension containing 5 mg of LPSNP and 1-phenylpiperazine functionalized LPSNP was stirred for 2 h at 500 rpm, after which it was drop-casted onto glass coverslips and left to dry. The resulting surface was used for contact angle measurements (OCA 15EC, Dataphysics, Germany), which were conducted using a fully automated measuring system equipped with a zoom lens and electronic syringe unit and were performed at ambient temperature. The contact angle measurements were taken by measuring the angles between the tangent line and the droplet on the sample surface, with both right and left angles being considered as a single measurement. All the materials were measured three times, and the average contact angle was calculated. A photo was taken for each measurement.

## Characterization

The physical and chemical properties of the nanoparticles were investigated using a series of techniques. Transmission electron microscopy (TEM) images were acquired using a HITACHI HT7700 (Tokyo, Japan) operated at 100 kV. To prepare the TEM samples, the nanoparticles were first dispersed in ethanol, and the resulting suspension was then deposited onto a carbon-coated copper grid and allowed to air-dry. The obtained TEM images were then analysed to investigate the size and shape of the nanoparticles. The scanning electron microscope (SEM), dark field scanning transmission electron microscopy (STEM), and the energy dispersive spectrometry (EDS) mapping images were obtained from HITACHI HF5000 Cs-STEM/TEM (Tokyo, Japan) operated at 200 kV. To prepare the samples, the nanoparticles were washed three times in ethanol, and the ethanol suspension was dropped onto a carbon-coated copper grid and allowed to air-dry. The surface area and pore size of the synthesized silica nanoparticles were measured using nitrogen adsorption–desorption at 77 K with a Micromeritics Tristar II 3020 system (Micrometrics Tristar II, United Kingdom). Before measurement, the samples (around 30 mg) were degassed on a vacuum pipeline at 393 K overnight to remove any adsorbed moisture or gases. The surface area of the nanoparticles was calculated using the Brunauer-Emmett-Teller (BET) method, which is widely used to determine the specific surface area of porous materials. Dynamic light scattering (DLS) and zeta potential measurements were performed in water at 25°C using a Malvern NanoZS setup (Malvern Panalytical, United Kingdom). DLS is a useful technique to measure the hydrodynamic diameter of nanoparticles in solution, which is often larger than the particle size measured by TEM due to the presence of a surface coating or adsorbed molecules. Zeta potential is a measurement of the surface charge of nanoparticles and provides important information about their stability and interactions with other molecules. Thermogravimetric analysis (TGA) was conducted in a temperature range of 25-900 °C at a heating rate of 10°C/min using a Mettler-Toledo setup (StarE, Switzerland). All the measurements were performed in triplicate for each sample, and the data are presented as mean ± standard deviation to ensure the reliability and accuracy of the results. Further analysis of the 1-phenylipiperazine functional group on LPSNP was conducted by Fourier Transform Infrared Spectroscopy (FTIR) using a PerkinElmer FTIR-ATR spectrometer.

Solid-state NMR experiments were performed on a Bruker Avance III spectrometer with a 300 MHz magnet equipped with a 4 mm double air bearing, magic angle spinning probe. The powdered samples were placed in a zirconia rotor with a Kel-F cap and rotated at 5 kHz. ^29^Si spectra were recorded with CPMAS and SP-hpdec pulse sequences, and the CPMAS data was used to confirm peak positions. For the CPMAS method, cross-polarization time was 5 ms, decoupling power 100 kHz. For SP-hpdec spectra the relaxation delay was 100 s, verified as sufficient for full relaxation. Decoupling power was 100 kHz as well. ^13^C CPMAS spectra used 1 ms CP time and 100 kHz decoupling power. Peak deconvolution and integration were carried out using SigmaPlot software.

## *In vitro* Mucus interaction

*In vitro* mucus interaction studies were conducted to assess the ability of the functionalized nanoparticles to penetrate and diffuse through a simulated mucus layer. To prepare the mucin-silica nanoparticle mixture, Mucin from porcine stomach was first dispersed in Milli-Q water to make a 10 mg/mL solution. The solution was then stirred at 300 rpm overnight to ensure complete dispersion. After centrifugation at 850 g for 0.5 h to remove excess insoluble mucin, pristine and 1-phenylpiperazine functionalized silica nanoparticles were added to the mucin solution with a concentration of 1 mg/mL The mixture was then incubated at 37°C with gentle stirring. At three specific time points (0, 30, and 180 min), samples were collected for particle size analysis using dynamic light scattering (DLS). This technique enabled the determination of the size distribution of the mucin-silica nanoparticle complex over time.

## *In vitro* Mucus penetration

Type II mucin isolated from pig stomach was employed to create the in vitro mucus models, as it is the primary component of intestinal mucus [2]. Mucus was simulated by dissolving 5% (w/v) mucin in PBS, sonicating, then applying to Transwell® permeable membrane supports to give a 2 mm deep layer. The supports were placed into a basal plate containing 1.5 mL of PBS in each well, and particle suspensions (2 mg/mL) were added to the apical surface of the mucus. Samples were taken from the basal wells over time with PBS replenishment, and the collected nanoparticles were digested with 1M NaOH and the silicon content was measured by ICP-MS to determine the fraction of particles transported across the barrier.

## Stability in simulated gut fluid

Fed-state simulated intestinal fluid (FeSSIF) was prepared by dissolving 4.071 g of FeSSIF buffer concentrate (Biorelevant) in 45.97 g of purified water, followed by pH adjustment to 5.0 using 1 M HCl or NaOH. FeSSIF (3F) powder (0.560 g) was subsequently added under continuous stirring until fully dissolved, and the final volume was adjusted to 50 mL with purified water. For stability assessment, nanoparticles were dispersed in freshly prepared FeSSIF at a concentration of 0.1 mg/mL and incubated at 37 °C. At predetermined time points, 1 mL aliquots were collected for dynamic light scattering (DLS) and polydispersity index (PDI) measurements.

## Cell culture

The Caco-2 and HT29-MTX -E12 were purchased from American Type Culture Collection (ATCC, Manassas, VA, USA). Caco-2 cells at passages 20-30 and HT29-MTX -E12 cells at passages 50-60 were used in the experiments. The cells were cultured in Dulbecco's Modified Eagle Medium (DMEM) supplemented with 10% fetal bovine serum (FBS), 1% Minimal Essential Medium (MEM), 1% (v/v) glutamine, 1% (v/v) sodium pyruvate solution, and 1% (v/v) penicillin/streptomycin (P/S). The cells were maintained under optimal growth conditions of 37°C, 5% CO_2_, and high humidity. Passage of cells was performed when the cells reached 80-90% confluence using 0.25% trypsin-EDTA.

## Cell viability

Caco-2 and HT29-MTX -E12 cells were cultured in Corning 96-well plates at a cell density of 1 x 10^4^ cells per well and allowed to grow for 24 h. After this initial incubation period, nanoparticles of various concentrations between 0.1 mg/mL and 2 mg/mL were added to the cells. The cells were then allowed to incubate for another 24 h, 3 days and 7 days. The control group, which consisted of cells only, was only exposed to cell culture medium. Following the 24-h incubation period, the cells were washed twice with PBS to remove any excess particles. After that, DMEM containing 10% MTS was added to the cells. The cells were then incubated for an additional 2 hours before the absorbance was read at 490 nm using a spectrophotometer (SpectroStar Nano, BMG Labtech).

Caco-2 and HT29-MTX -E12 cells were cultured in a 4-chamber 35 mm glass bottom dish with a 20 mm microwell for 24 h before confocal microscopy analysis. The cell density per well was 1 x 10^5^ cells. Afterward, nanoparticles at a concentration of 2 mg/mL were dispersed and added to the cells, which were then cultured for an additional 24 h. Subsequently, the cells were fixed with 4% Paraformaldehyde (PFA) at room temperature for 20 min to prepare them for confocal microscopy imaging. For staining, the cells were rinsed with ice-cold PBS and permeabilized with 0.2% Triton-X100 for 5 minutes. They were then incubated with a solution containing Alexa Fluor™ 488 Phalloidin, following the manufacturer's instructions, for 30 minutes at room temperature. Finally, the cells were stained with DAPI (4′,6-diamidino-2-phenylindole, 3µM) for 5 min at room temperature. Images were captured using a laser scanning confocal microscope, specifically the Olympus FV3000, at a magnification of 40X.

## Transepithelial transport effect of silica nanoparticles

Caco-2 monolayer was established on the 12-well Transwell (Corning). The Transwell inserts were coated with rat tail collagen I with 5 µL/cm [3, 4]. Then the Caco-2 cells were seeded on the apical chamber of Transwell at the density of 2 × 10^5^ cells/well. The cell culture medium was composed of DMEM added with 1% P/S, MITO^+^ Serum extender. The cells were cultivated for 2 days. After 2 days, the medium was replaced with EDM which contains DMEM medium with MITO^+^ Serum extender and 2 mM sodium butyrate. The cells were cultivated for another 2 days before the experiment. The transepithelial electrical resistance (TEER) values were recorded daily by EVOM volt-ohmmeter (World Precision Instruments, Sarasota, FL, USA).

Caco-2/HT29-MTX Co-culture monolayer was also established on the 12-well Transwell. The Caco-2 and HT29-MTX -E12 cells were plated in the Transwell chamber with 1× 10^5^ cells/well at the ratio of 9:1. The cells were cultured for 8 days, and the culture medium was replaced every other day.

The transwells with over 150 Ω*cm^2^ TEER value were selected for further experiments, representing physiologically relevant barrier integrity consistent with established models [2, 4]. Nanoparticles were dispersed in 0.5 mg/mL insulin solution in Hanks’ Balanced Salt Solution (HBSS) to form a suspension. The suspension was added into the apical chamber. The basolateral compartment was added with a mixture of 1.2 mL HBSS and 0.3 mL HEPES buffer. The TEER value was measured at the time-points of 0.5, 1, 2, and 3 h, with 25 µL basolateral solution taken and replaced with fresh HBSS to evaluate the insulin transport amount by using manufacturer’s instructions on a BCA kit. After 3 h treatment, silica nanoparticles were removed, and TEER was recorded for recovery in DMEM medium after 4, 6, and 24 h.

The apparent permeability coefficient (P_app_, cm s^-1^ ) was determined according to the following equation:

$$Papp=(\frac{dQ}{dt})(\frac{1}{A\cdot C_{0}})$$

where dQ is the insulin concentration in the basolateral chamber, dt is the treatment time. A is the surface area of the cell monolayer (1.12 cm^2^), and C_0_ is the initial insulin concentration in the apical chamber.

## Tight junction staining

Caco-2 monolayer and Caco-2/HT29-MTX Co-culture monolayer was established as described before. To investigate the change of tight junction, transwell filters with Caco-2 monolayers and Caco-2/MTX-HT29 Co-culture monolayer were collected for confocal microscopy after 3 h treatment to stain the tight junctions between cells. They were rinsed with ice cold PBS to remove excess silica nanoparticles after 3 h treatment. Then the cells were fixed with 4% PFA at room temperature 15 min. After rinsing with PBS 3 times, they were permeabilized with 0.3% Triton-X100 for 5 min and 1% BSA solution for 1 h at room temperature to avoid the non-specific antibody binding. Next, the cells were incubated in Alexa Fluor 555 conjugated anti- Zonula occludens-1 (ZO-1) antibodies (20 µg/mL) at 4 °C overnight. The cells were washed with PBS and finally stained by Hoechst for 5 min at room temperature and then mounted on glass slides. Images were captured using Laser scanning confocal microscope Leica SP8 at 40X magnification.

## Animal study

## Six-week-old C57BL/6 mice were purchased from the Ozgene ARC (WA, Australia). The mice were housed under standard laboratory conditions, with 12-h light/dark cycle, 25 ± 2°C temperature, 55-60% relative humidity, in a conventional clean, Helicobacter hepaticus-free facility. Animals were either fed a standard chow diet (NCD; Speciality feeds Australia, SF00- 100) or a high fat diet (HFD, Speciality feeds Australia, SF04-001, containing 46% of available energy as saturated fat, 20% protein, and 4.80% crude fibre) and had access to water *ad libitum*. All experimental protocols and procedures conducted were complied with the the National Health and medical research council guidelines at the UQ-TRI BRFs guidelines and approved by the Institutional Animal Ethics Committee of the University of Queensland (2022/AE000017).

## Intestinal permeability to dextran

Following 10 h fasting, the animals were orally administered with 100 mg/kg of the pristine or 1-phenylpiperazine functionalized silica nanoparticle suspension (at a concentration of 10 mg/mL in water). After a 2 h interval, the mice were gavaged FITC-dextran 4 kDa in water at a dosage of 60 mg/kg. Three hours following the administration, blood samples were collected and subjected to centrifugation to separate the serum. The concentration of FITC in the serum was determined by measuring the fluorescence intensity using a plate reader, with an excitation wavelength of 485 nm and an emission wavelength of 515 nm. The obtained fluorescence values were then compared to a calibration curve specific to each experiment.

## Capsule preparation

Oral insulin capsule (675 U kg^−1^) preparation was followed by reported method [2]. Briefly, insulin from the bovine pancreas was mixed with protease inhibitor aprotinin and de-activated bovine serum albumin (BSA) with a 3:1:1 ratio in an aqueous solution and then freeze-dried. Size M capsules for large mice (> 30 g) were filled with 1.5-2.0 mg filler and the exact weights were recorded. The filled capsules were dip-coated with 7% Eudragit L100-55 solution in ethanol. The coating was repeated 3 times and air-dried after each coating.

## Insulin release

Size M capsules for large mice were filled with ~2.0 mg insulin and the exact weights were recorded. The filled capsules were dip-coated with 7% Eudragit L100-55 solution in ethanol. The coating was repeated 3 times and air-dried after each coating. For the release, capsules were incubated under 37 °C with gentle stirring at pH 1.9 and pH 7.4 PBS buffer. 1 capsule in ~2 mL solution (based on insulin weight, 1 mg/mL). After 30min, 1h, 2h, 4h and 6h, 100 ul solution (~5% of total volume) was taken out and replace with fresh buffer. The protein content was measured by BCA kit with manufacture instructions.

## Glucose tolerance test and insulin tolerance test

Mice were fed with high fat diet (HFD) for 12 weeks to develop hyperglycaemia, and normal diet control mice were fed with normal chow. The mice were tested for glucose tolerance (GTT) and insulin tolerance (ITT) a week before the experiment to investigate the base line. For the GTT, 5 mice with normal chew diet and 5 mice with high fat diet was randomly selected and intra-peritoneal (IP) injected with 20% glucose (100 µL/mice). After injection, the blood glucose level was measured by taking a drop of blood from the tail at 15 min, 30min, 60min and 120min. For ITT, 5 mice with normal chow diet and 5 mice with high fat diet were randomly selected and intra-peritoneal (IP) injected with 0.75 U/kg insulin Humalog. After injection, the blood glucose level was measured by taking a drop of blood from the tail at 15 min, 30min, 60min and 120min.  If the blood glucose level of mice was lower than 2.5 mmol/L during the experiment, 100 µL 20% glucose was injected to rescue the mice.

## Oral insulin delivery with capsules

In this experiment, the mice were fed a high-fat diet for 12 weeks before the experiment to induce hyperglycaemia. Before the experiment, the large mice (> 30g) were orally gavaged with PBS (controls, 10 µL/g) or 100 mg kg^−1^ large pore silica nanoparticles or 1-phenylpiperazine functionalized silica nanoparticles. Then, 5 mg kg^−1^ metoclopramide hydrochloride was injected subcutaneously to stimulate gastric emptying. After 2 h, the insulin capsules were orally gavaged into mice and immediately flushed with PBS or 100 mg kg^−1^ large pore silica nanoparticles or piperazine functionalized silica nanoparticles. The blood glucose levels were measured at 0.5, 1, 2, 4, 6, 8 and 10 h.

## Histology

The organs (Small intestine, colon, kidney, liver and pancreas) from sacrificed mice were collected and fixed 24h in 10% neutral buffered formalin (NBF) and then transferred to 70% ethanol for embedding and sectioning. The paraffin sections were prepared and stained with hematoxylin and eosin for histological examination.

## Statistical analysis

All data are presented as mean ± standard deviation (SD) unless otherwise specified. Statistical analyses were performed using GraphPad Prism 9.0 software from GraphPad software in California, USA. For comparisons among more than two groups with a single independent variable, one-way analysis of variance (one-way ANOVA) was used, followed by an appropriate post hoc multiple comparisons test (Tukey’s test) to determine differences between individual groups. For experiments involving two independent variables, two-way analysis of variance (two-way ANOVA) was performed to evaluate the main effects of each factor as well as their interaction. When significant effects were detected, post hoc multiple comparisons testing (Tukey’s test) was applied. Nanoparticle sizes were analyzed using Image J software. The reported nanoparticle size values were based on the mean of at least 30 individual particle sizes from 2 to 3 TEM images.


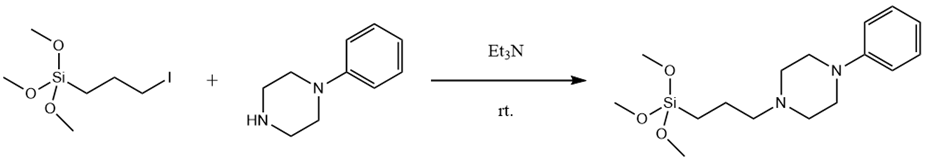


Figure S1. Scheme for synthesis of 1-phenyl-4-(3-(trimethoxysilyl)propyl)piperazine. Et_3_N: triethylamine and rt: room temperature.


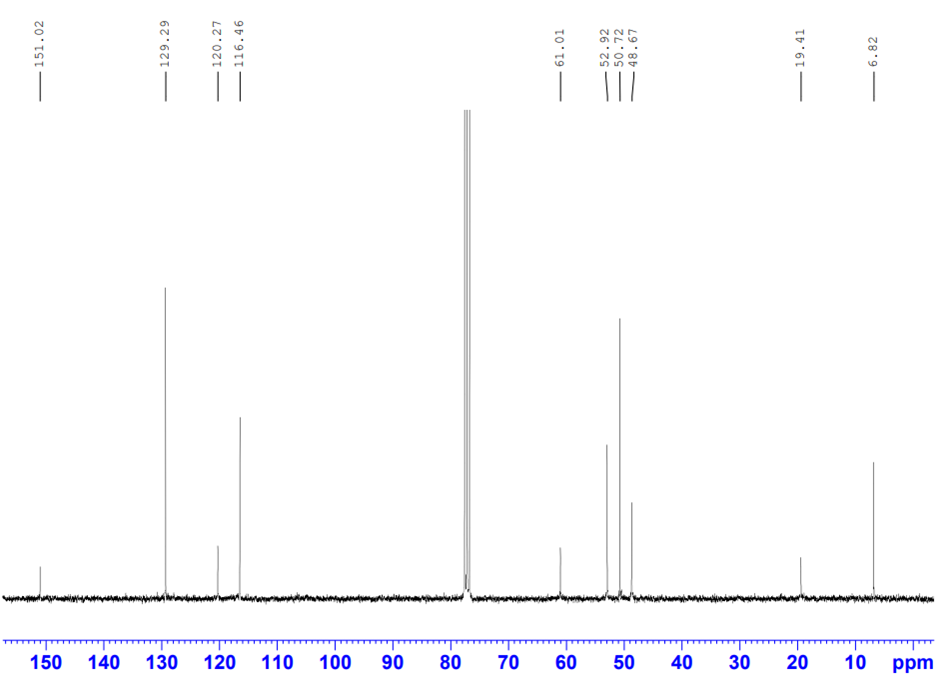


Figure S2. ^13^C NMR spectrum of 1-phenyl-4-(3-(trimethoxysilyl)propyl)piperazine.


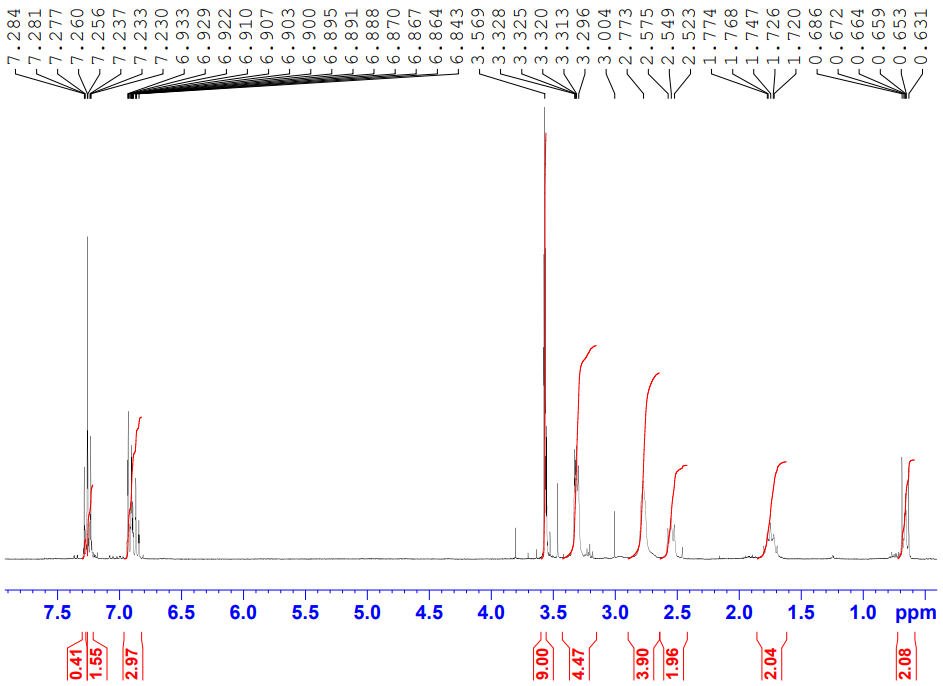


Figure S3. ^1^H NMR spectrum of 1-phenyl-4-(3-(trimethoxysilyl)propyl)piperazine.


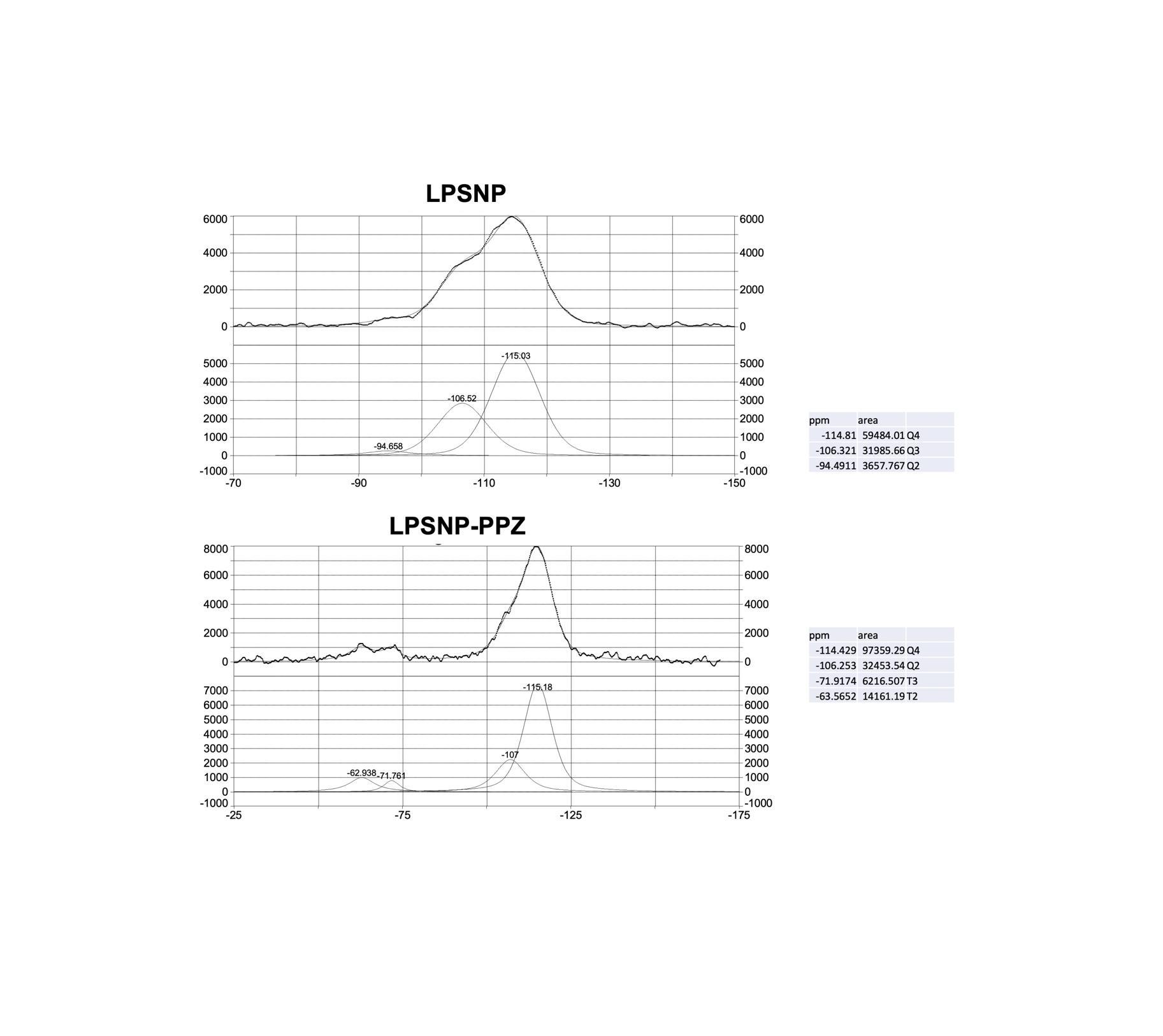


Figure S4. Solid-state ^29^Si NMR spectrum of LPSNP and LPSNP-PPZ nanoparticles.

Table S1. Solid-state ^29^Si NMR chemical shifts and related area

| LPSNP | | | LPSNP-PPZ_H_ | | |
| --- | --- | --- | --- | --- | --- |
|  | ppm | area |  | ppm | area |
| Q2 | -94.4911 | 3657.767 | **Q2** | -106.253 | 32453.54 |
| Q3 | -106.321 | 31985.66 | **Q4** | -114.429 | 97359.29 |
| Q4 | -114.81 | 59484.01 | **T2** | -63.5652 | 14161.19 |
|  |  |  | **T3** | -71.9174 | 6216.507 |


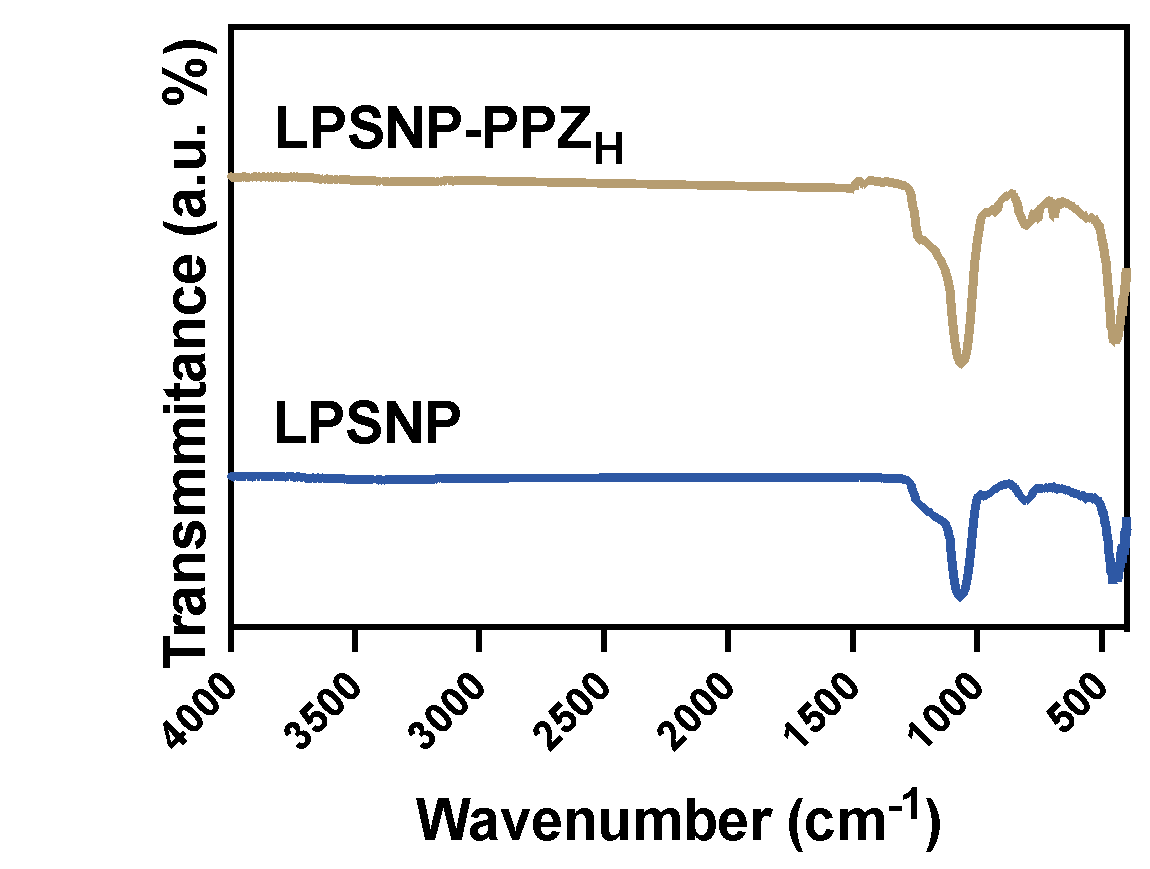


Figure S5. FTIR spectrum of LPSNP and LPSNP-PPZ nanoparticles.

Table S2. CHN elemental analysis

| Element (%) | | LPSNP | LPSNP-PPZ_H_ |  |
| --- | --- | --- | --- | --- |
| C | 0.10 | | 4.76 | |
| H | 0.39 | | 1.43 | |
| N | 0 | | 1.65 | |


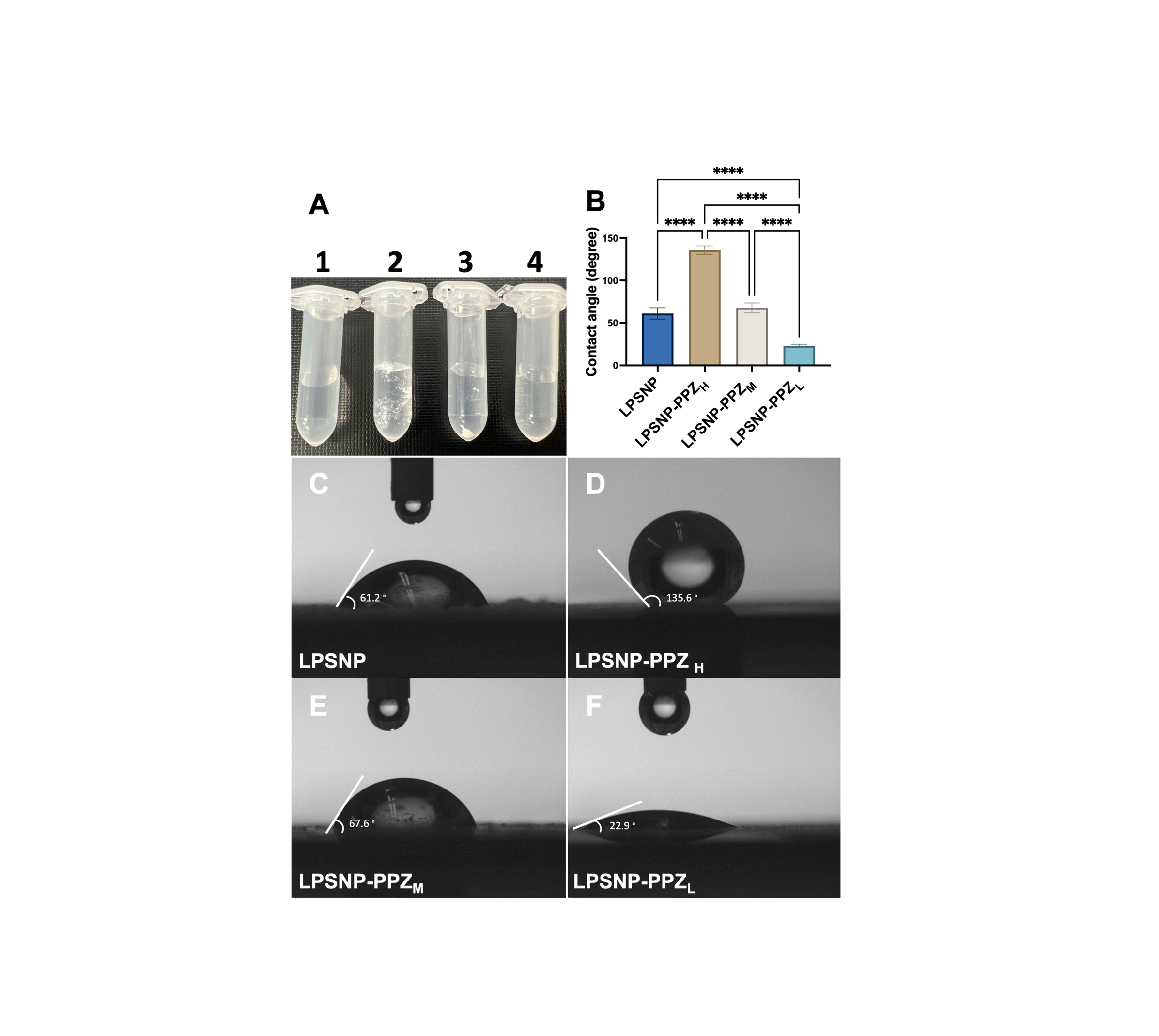


Figure S6. The high density of 1-phenylpiperazine functionalization increased the hydrophobicity. (A) Nanoparticle suspension in water (2 mg/mL), 1. LPSNP; 2. LPSNP- PPZ_H_; 3. LPSNP- PPZ_M_; 4. LPSNP-PPZ_L_. (B). The contact angle of LPSNP and LPSNP with different functionalization densities of 1-phenylpiperazine. (C-F). The contact angle photos of LPSNP and LPSNP with different functionalization densities of 1-phenylpiperazine. ****P< 0.0001, n=3, mean ± SD.


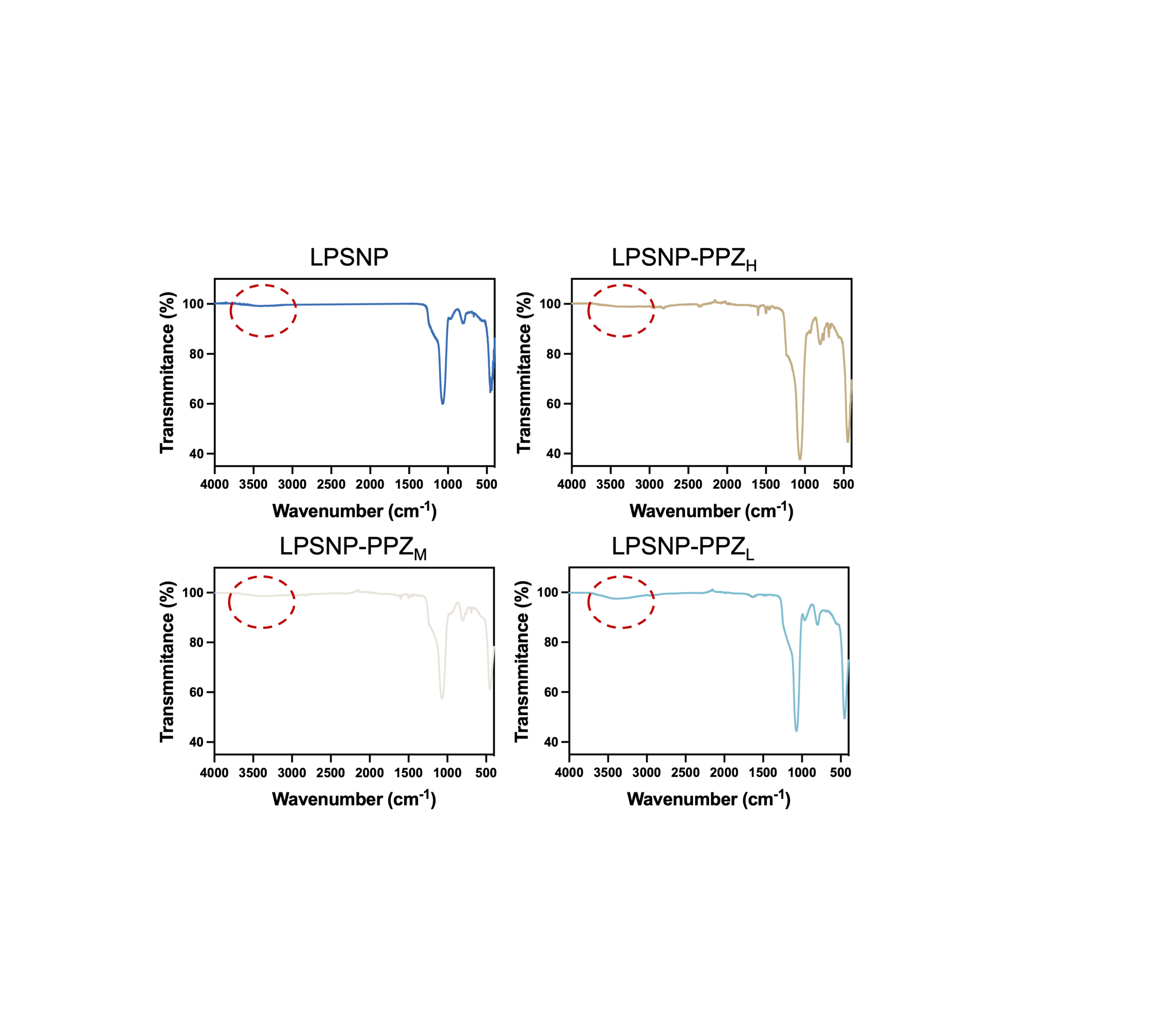


Figure S7. FTIR spectrum of LPSNP, LPSNP-PPZ_H_, LPSNP-PPZ_M_, and LPSNP-PPZ_L._

Table S3. Weight loss, functional molecule grafting density, and surface coverage of the silica nanoparticles calculated from TGA and BET data

|  | TGA wt loss 25-150°C  (wt %) | TGA wt loss 150-900°C  (wt %) | Organic content  (wt %) | PPZ grafting density (mmol/g)^a^ | PPZ surface coverage (molecules/nm^2^)^b^ |
| --- | --- | --- | --- | --- | --- |
| LPSNP | 5.9 | 3.1 | 0 | 0 | 0 |
| LPSNP-PPZ_L_ | 4.4 | 11.3 | 8.2 | 0.40 | 0.38 |
| LPSNP-PPZ_M_ | 2.4 | 19.5 | 16.4 | 0.81 | 0.77 |
| LPSNP-PPZ_H_ | 2.3 | 24.8 | 21.7 | 1.07 | 1.01 |

^a^Calculated from the organic content (wt %) and the MW of the 3-(4-phenylpiperazin-1-yl)propyl group (203.309 g/mol). ^b^Calculated from the corresponding grafting density (mmol/g) and the BET surface area of LPSNP (6.34 × 10^20^ nm^2^/g).


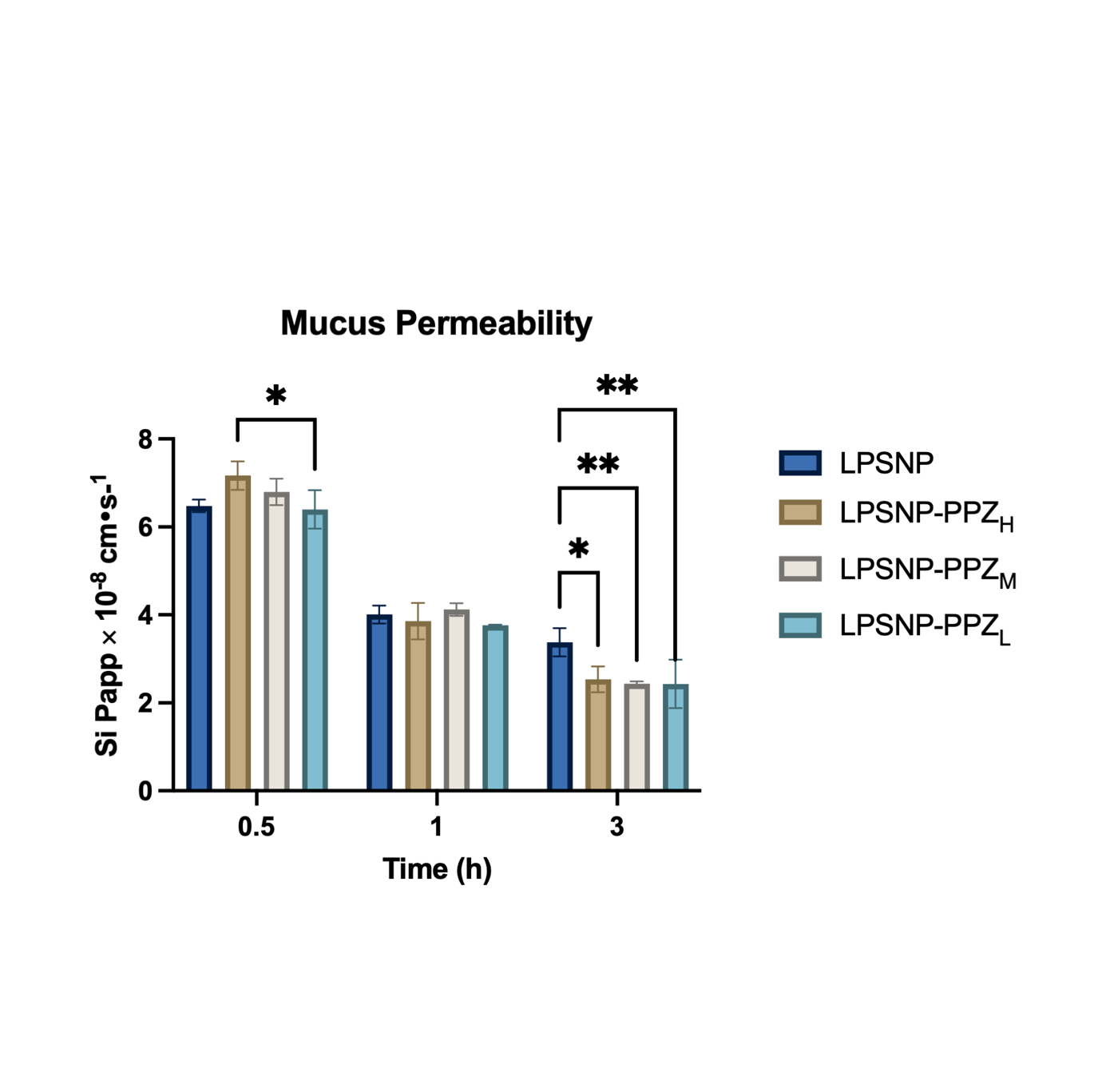


Figure S8. [1]. Apparent permeability coefficients (Papp) of silicon (Si) in silica nanoparticles across a mucus layer were quantified for unmodified LPSNP and PPZ-modified particles (LPSNP–PPZ_H_, LPSNP–PPZ_M_, LPSNP–PPZ_L_ at 0.5, 1, and 3 h. n=3, mean + SEM, *p<0.05, **p<0.01.


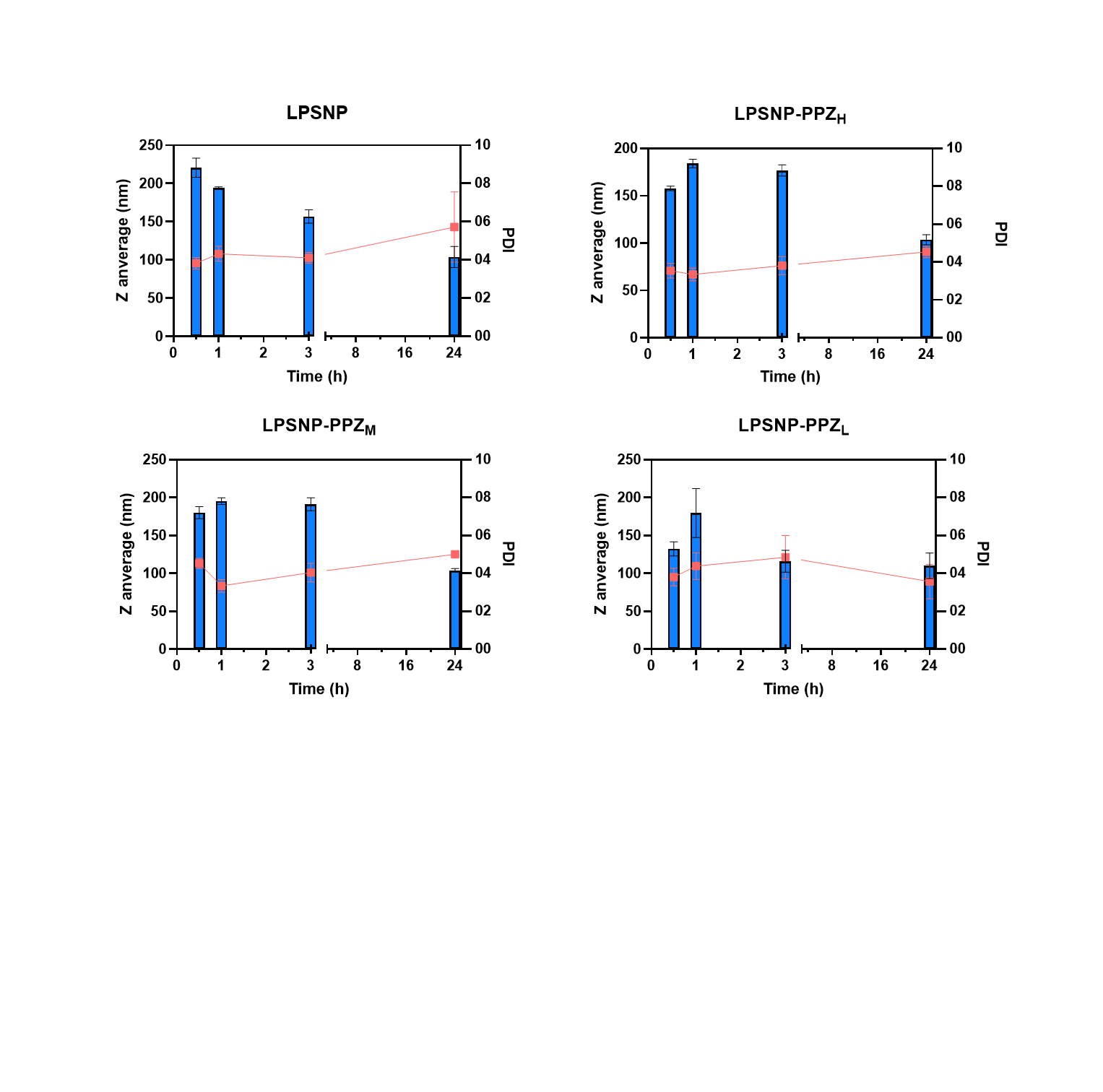


Figure S9. Colloidal stability of LPSNP and LPSNP-PPZ formulations in simulated intestinal fluid. Time-dependent changes in hydrodynamic diameter (Z-average, blue bars, left y-axis) and polydispersity index (PDI, red line, right y-axis) for LPSNP, LPSNP-PPZH, LPSNP-PPZM, LPSNP-PPZL, over 24 h incubation at 37 °C. Data are presented as mean ± SD (n = 3).


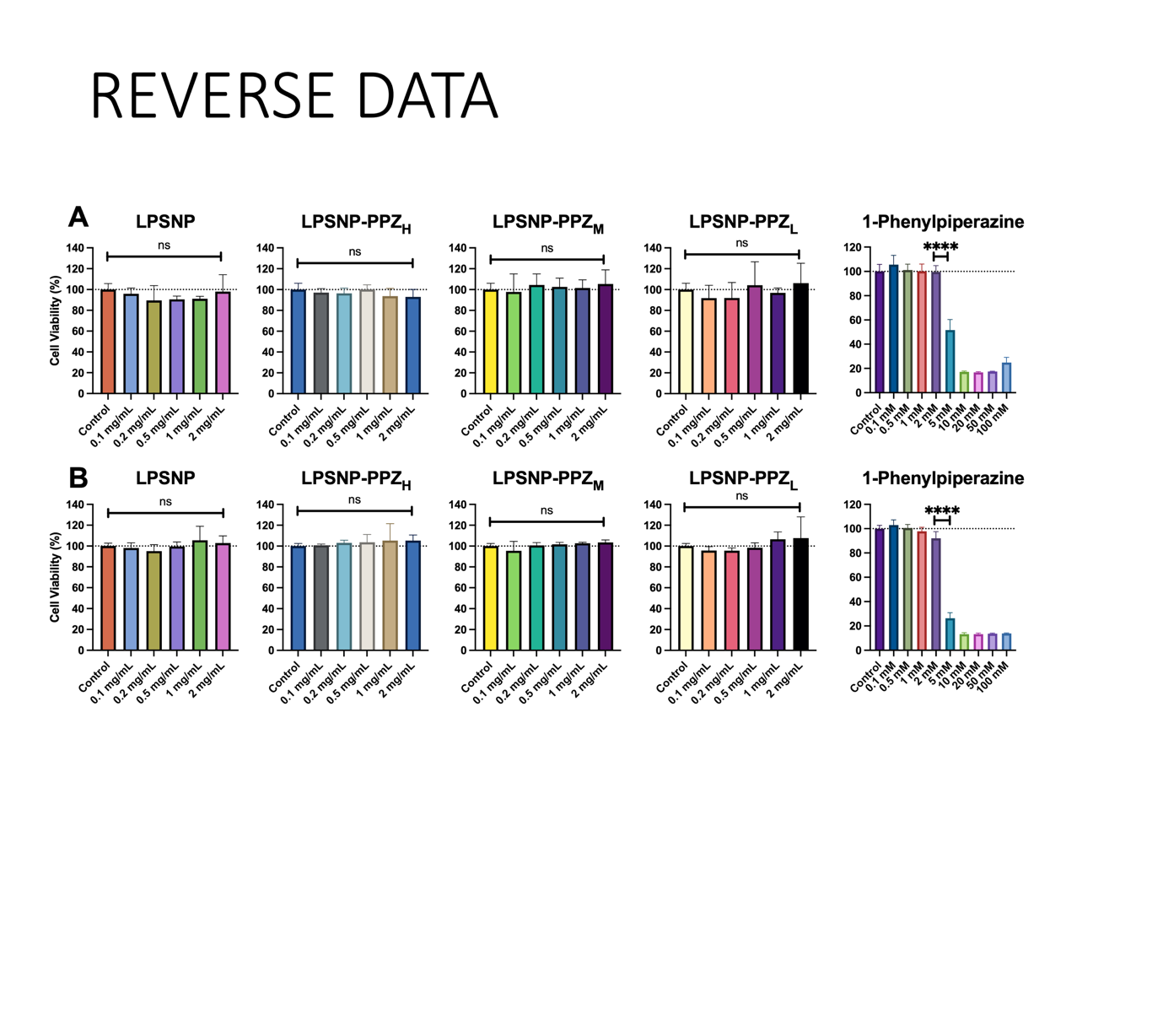


Figure S10. Cell viability of LPSNP, LPSNP-PPZ_H_, LPSNP-PPZ_M_, LPSNP-PPZ_L_ and 1-phenylpiperazine solution with different concentrations were tested in (A) Caco-2 cells and (B) MTX-HT29 cells. (*****P* < 0.001). n=4, mean + SD.


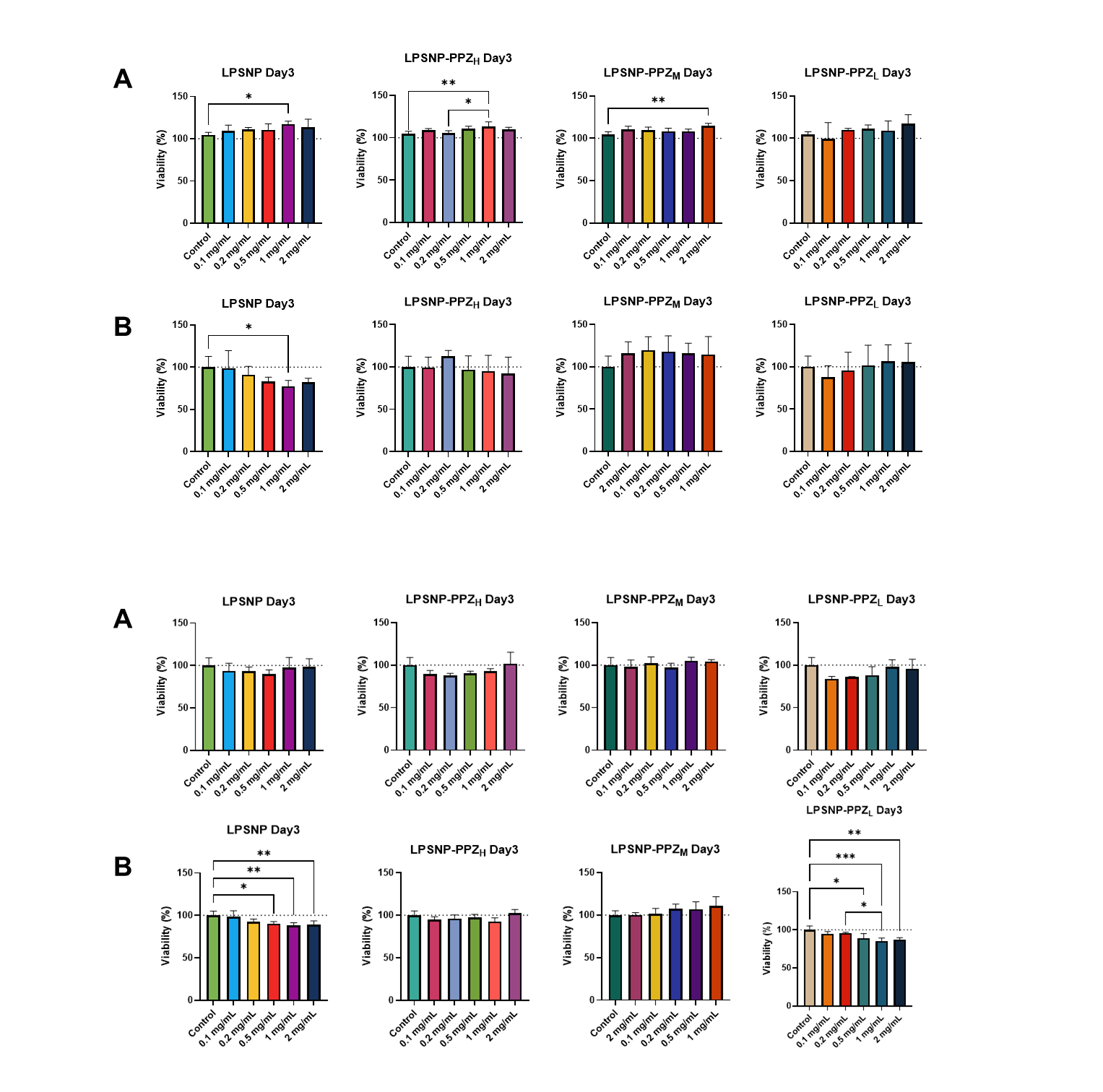


Figure S11. Cell viability of LPSNP, LPSNP-PPZ_H_, LPSNP-PPZ_M_, LPSNP-PPZ_L_ with different concentrations were tested in (A) Caco-2 cells and (B) MTX-HT29 cells at day 3. (*P < 0.05, **P < 0.01). n=4, mean + SD.


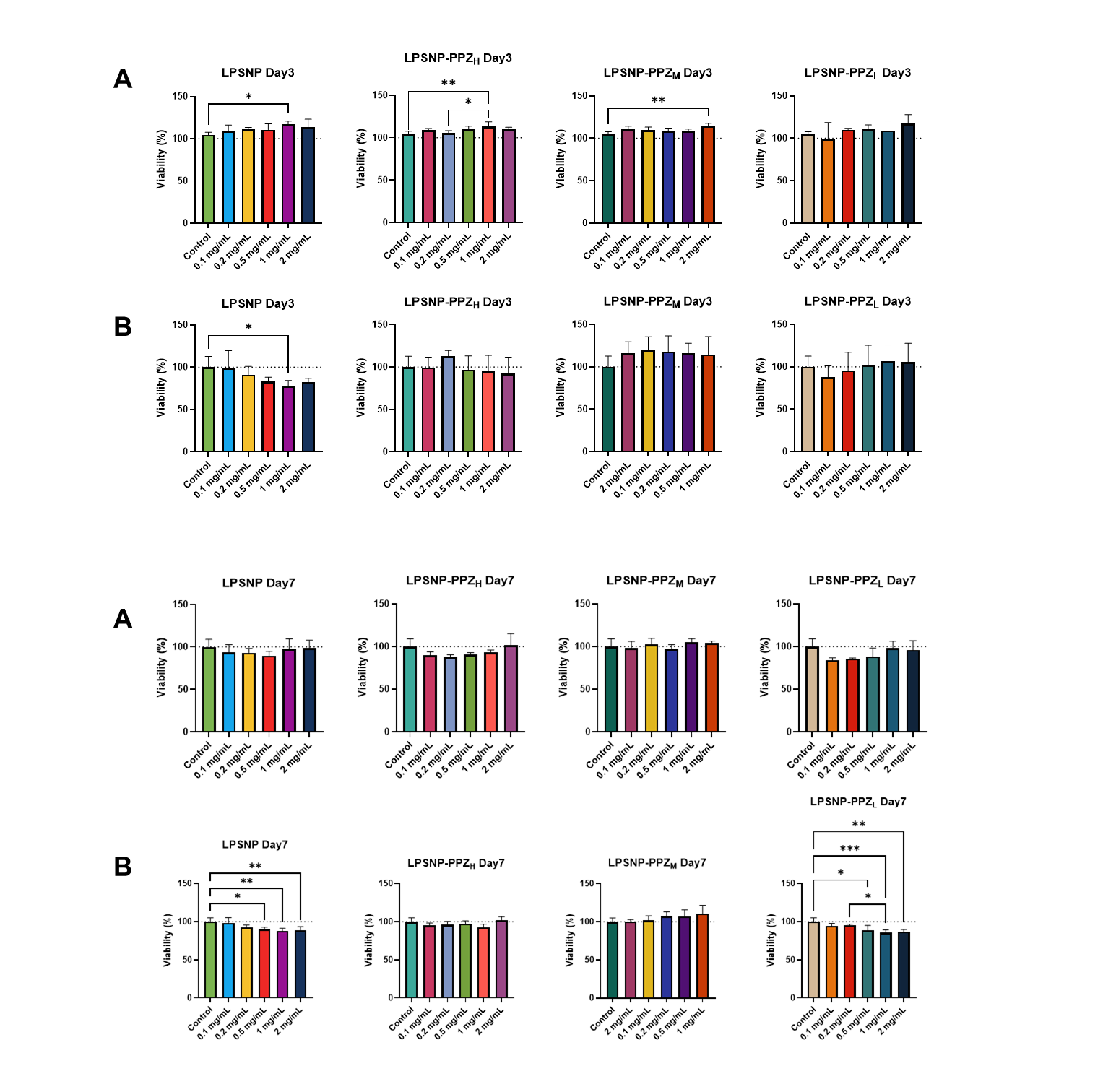


Figure S12. Cell viability of LPSNP, LPSNP-PPZH, LPSNP-PPZM, LPSNP-PPZL with different concentrations were tested in (A) Caco-2 cells and (B) MTX-HT29 cells at day 7. (*P < 0.05, **P < 0.01, ***P < 0.001). n=4, mean + SD.


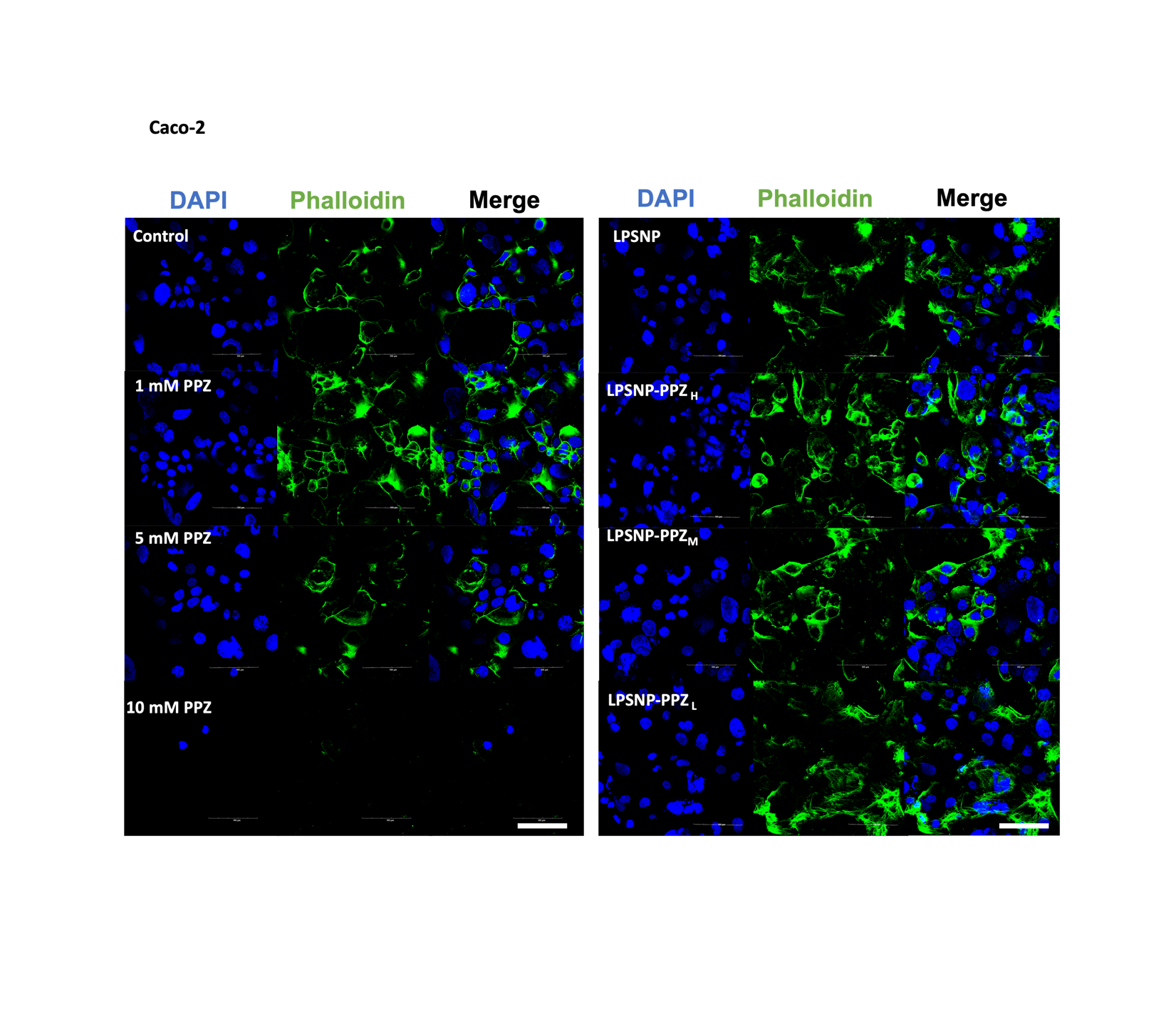


Figure S13. Representative confocal images of Caco-2 cells with 24 h treatment of 1 mM, 5 mM and 10 mM of 1-phenylpiperazine, pristine silica nanoparticles LPSNP, and 1-phenylpiperazine grafted LPSNP-PPZ_H_, LPSNP-PPZ_M,_ LPSNP-PPZ_L_ (2 mg/mL). Scale Bar: 100 µm.


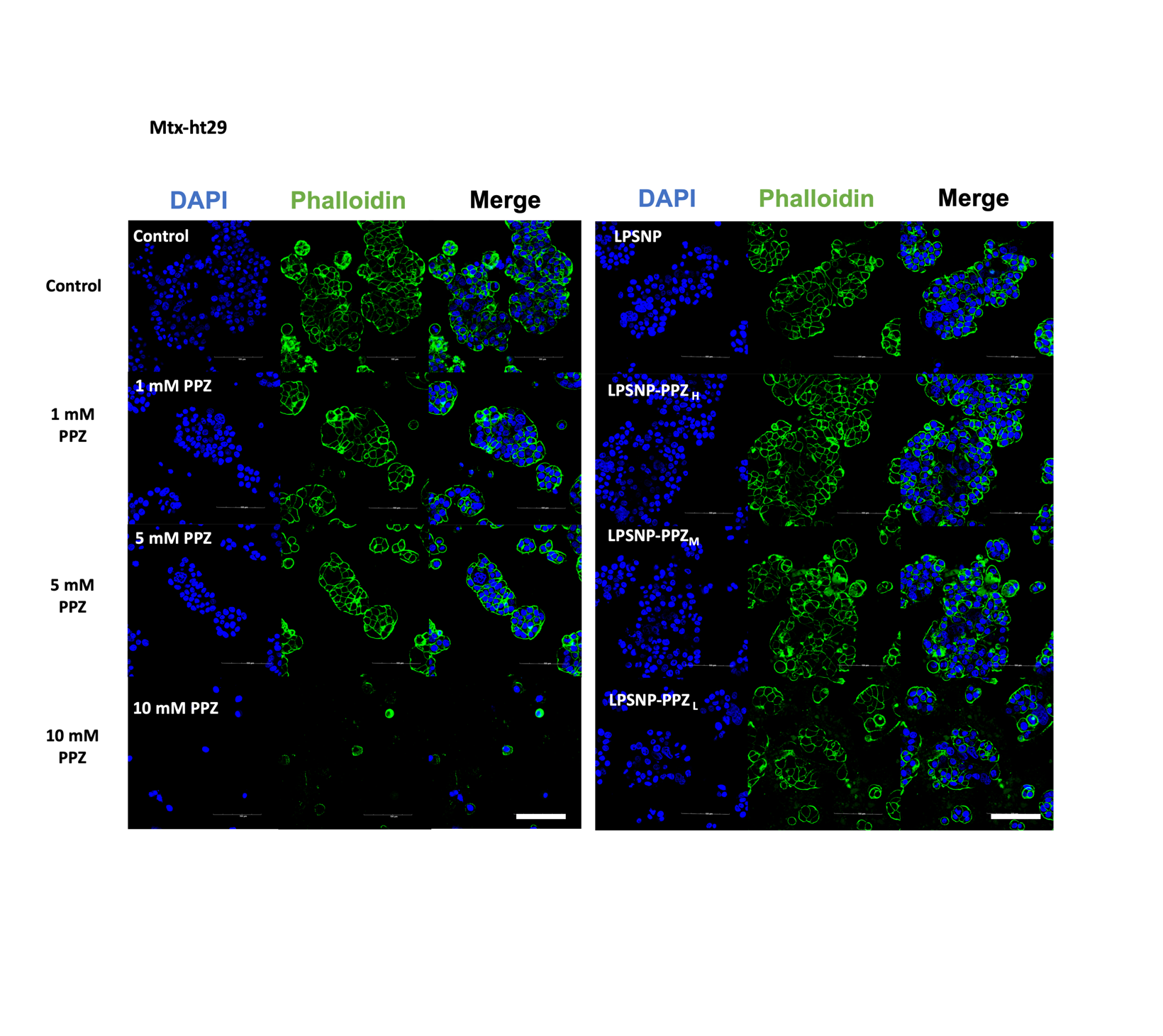


Figure S14. Representative confocal images of MTX-HT29 cells with 24h treatment of 1 mM, 5 mM and 10 mM of 1-phenylpiperazine, pristine silica nanoparticles LPSNP, and 1-phenylpiperazine grafted LPSNP-PPZ_H_, LPSNP-PPZ_M,_ LPSNP-PPZ_L_ (2 mg/mL). Scale Bar: 100 µm.


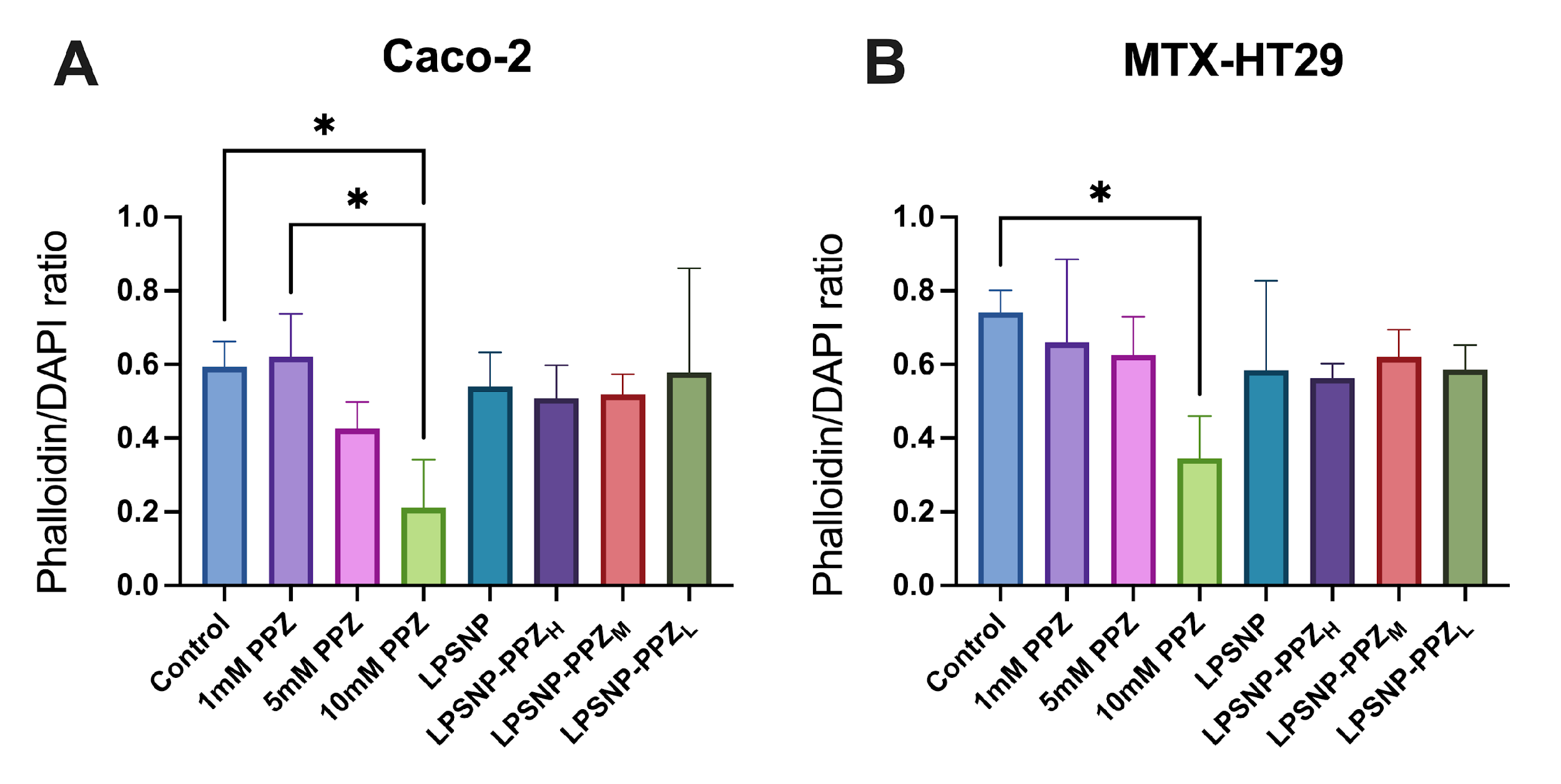


Figure S15. Quantification of Phalloidin/DAPI fluorescent signal ratio in Caco-2 cells (A) and MTX-HT29 cells (B). Three images of each group were used for analysis with image J. n=3, mean+ SD, *p<0.05.


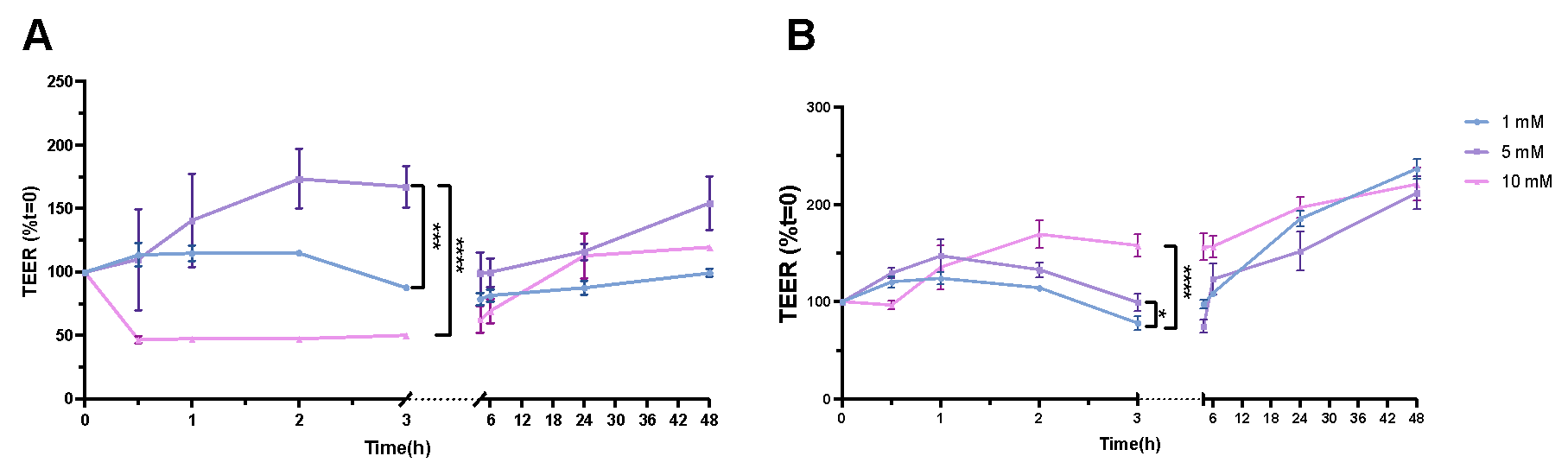


Figure S16. Effect of different concentrations of 1-phenylpiperazine solution on in vitro (A) Caco-2 monolayers and (B) Caco-2/MTX-HT29 co-culture monolayers. n=3, mean± SD,p<0.05, ***p<0.001, ****p<0.0001.


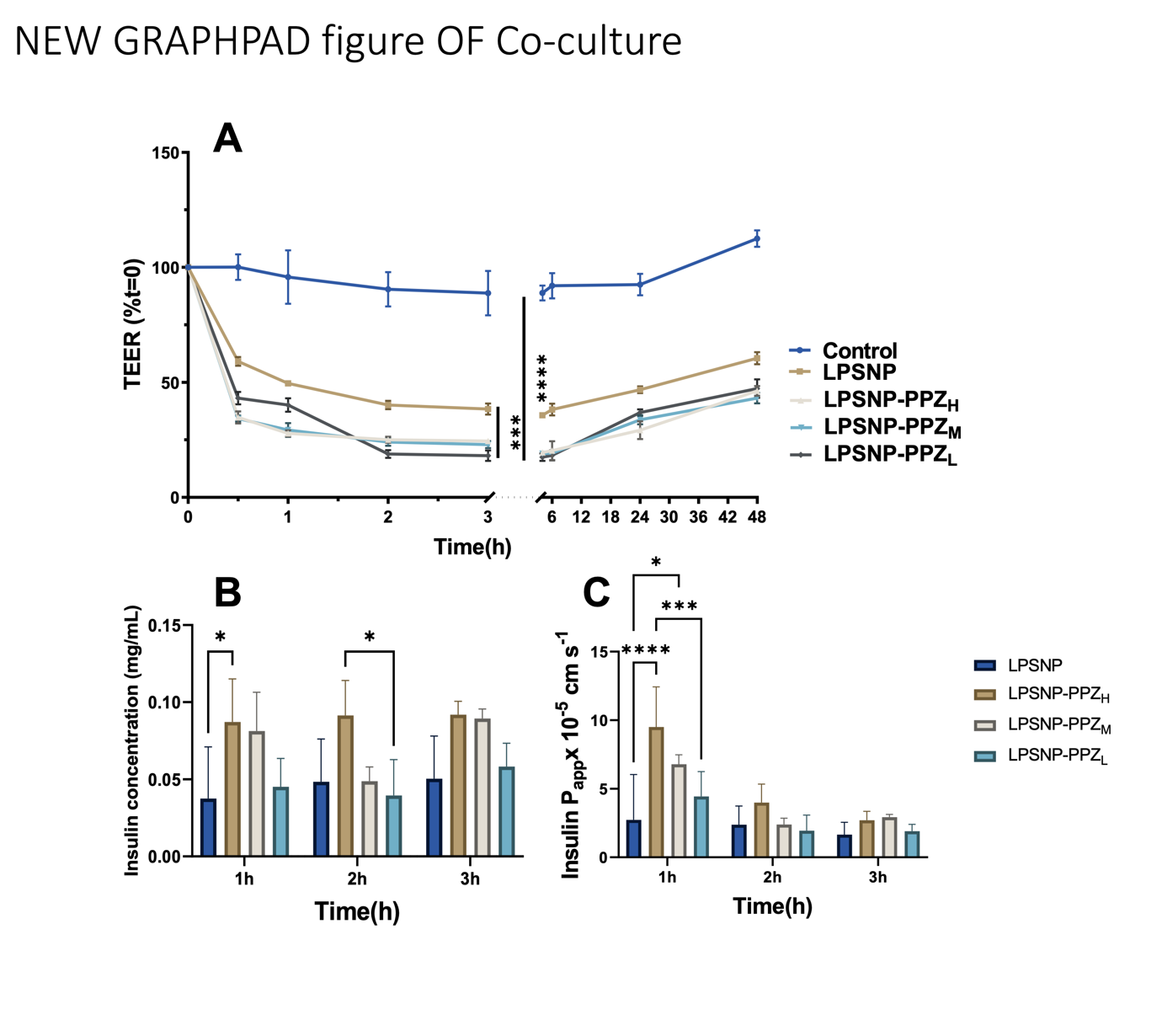


Figure S17. TEER value change of different silica nanoparticles (2 mg/mL) on *in vitro* Co-culture models (A). The concentration of insulin in the basolateral chamber and their related Papp value (B) and (C), respectively. The data was subtracted from insulin permeation amount in the control group (Co-culture monolayer with insulin alone). n=3, mean + SEM, *p<0.05, ***p<0.001, ****p<0.0001.


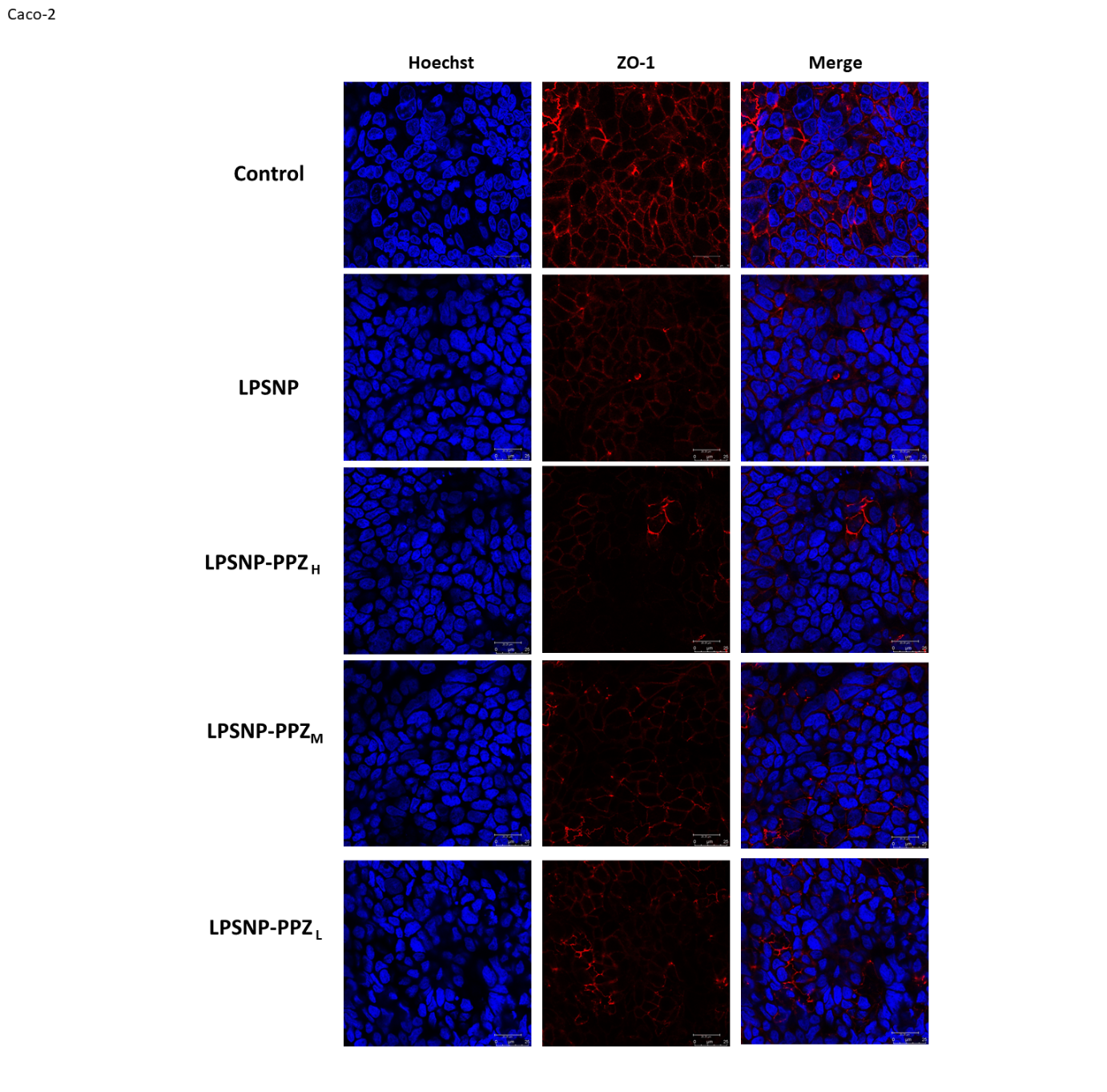


Figure S18. Representative confocal images of tight junction after PPZ particles applied to Caco-2 monolayer model. The tight junction protein Zonula occludens-1 (ZO1) was stained in red, and the nuclei is in blue. n=3. Scale Bar: 20 µm.


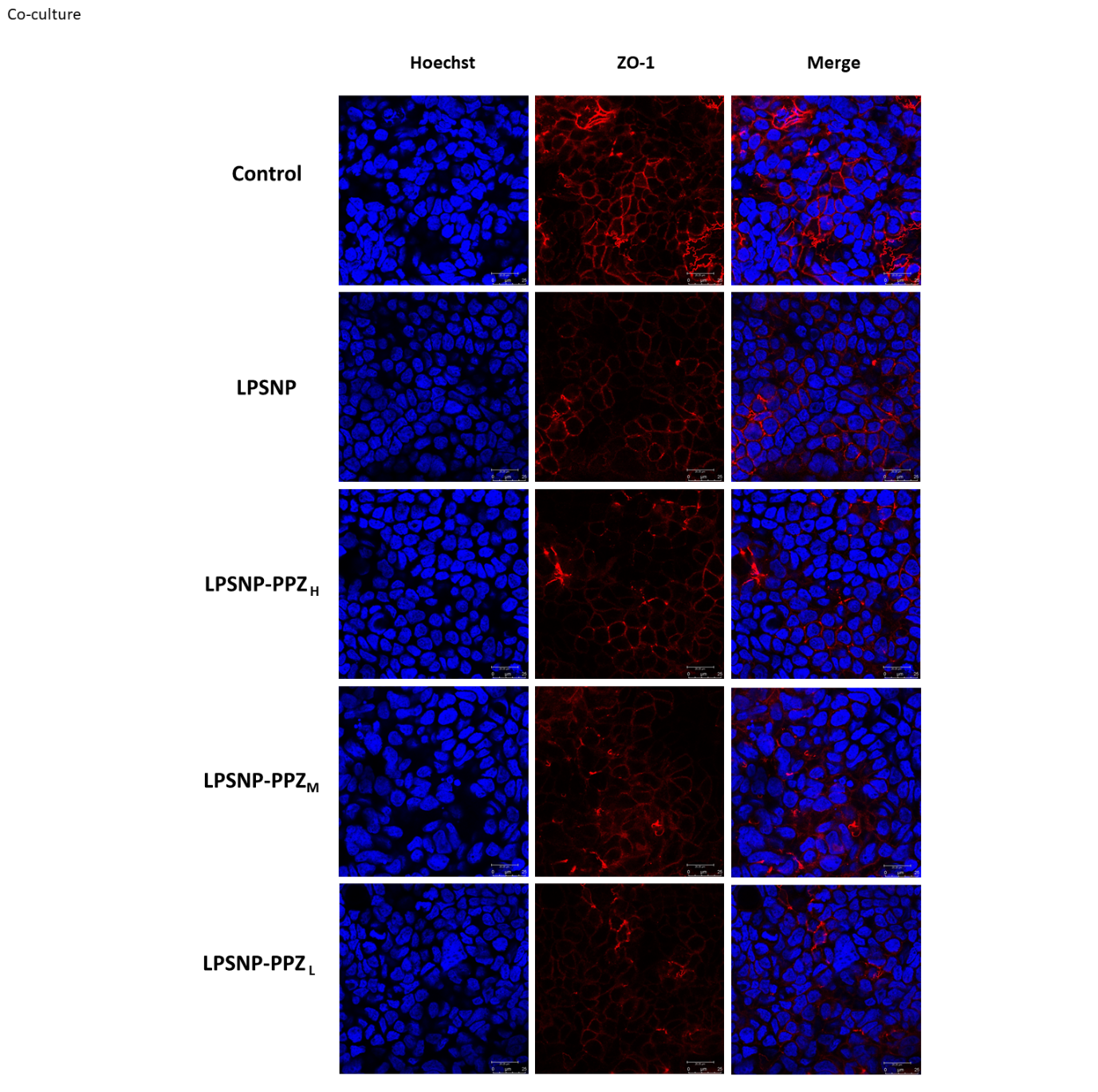


Figure S19. Representative confocal images of tight junction after PPZ particles applied to co-culture model. The tight junction protein Zonula occludens-1 (ZO1) was stained in red, and the nuclei is in blue. n=3. Scale Bar: 20 µm.


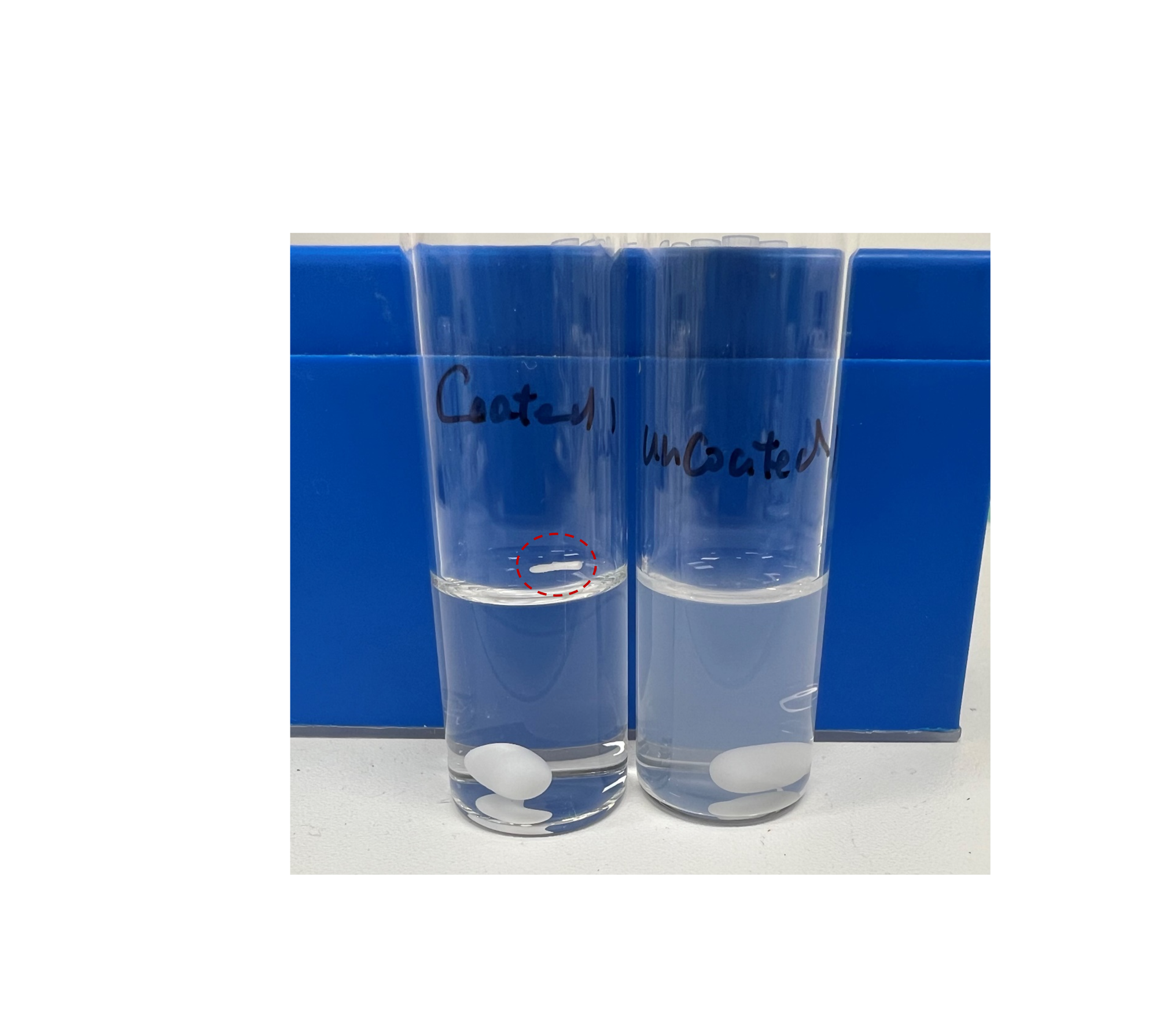


Figure S20. The Eudragit L100-55 coated size M mice capsule and uncoated capsule were gently stirred under pH 1.9 buffer for 30 min. The coated capsule was still sealed (circled in red) while the uncoated capsule dissolved and released drugs.


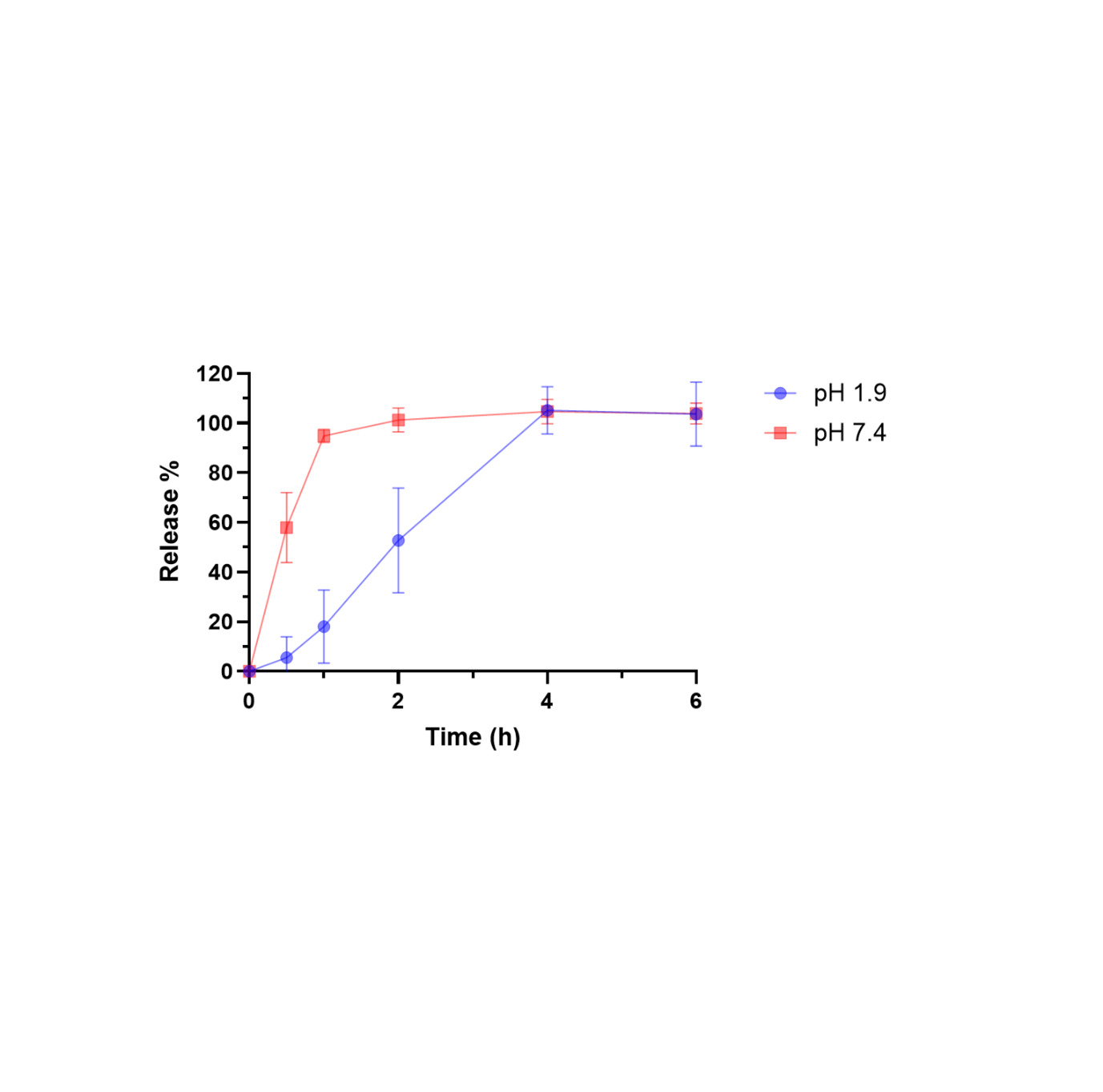


Figure S21. Insulin release profiles from Eudragit-coated insulin capsules under gastrointestinal conditions. Cumulative insulin release (%) was measured at pH 1.9 (simulated gastric fluid, blue circles) and pH 7.4 (simulated intestinal fluid, red squares) over 6 h. n=3, mean + SD.


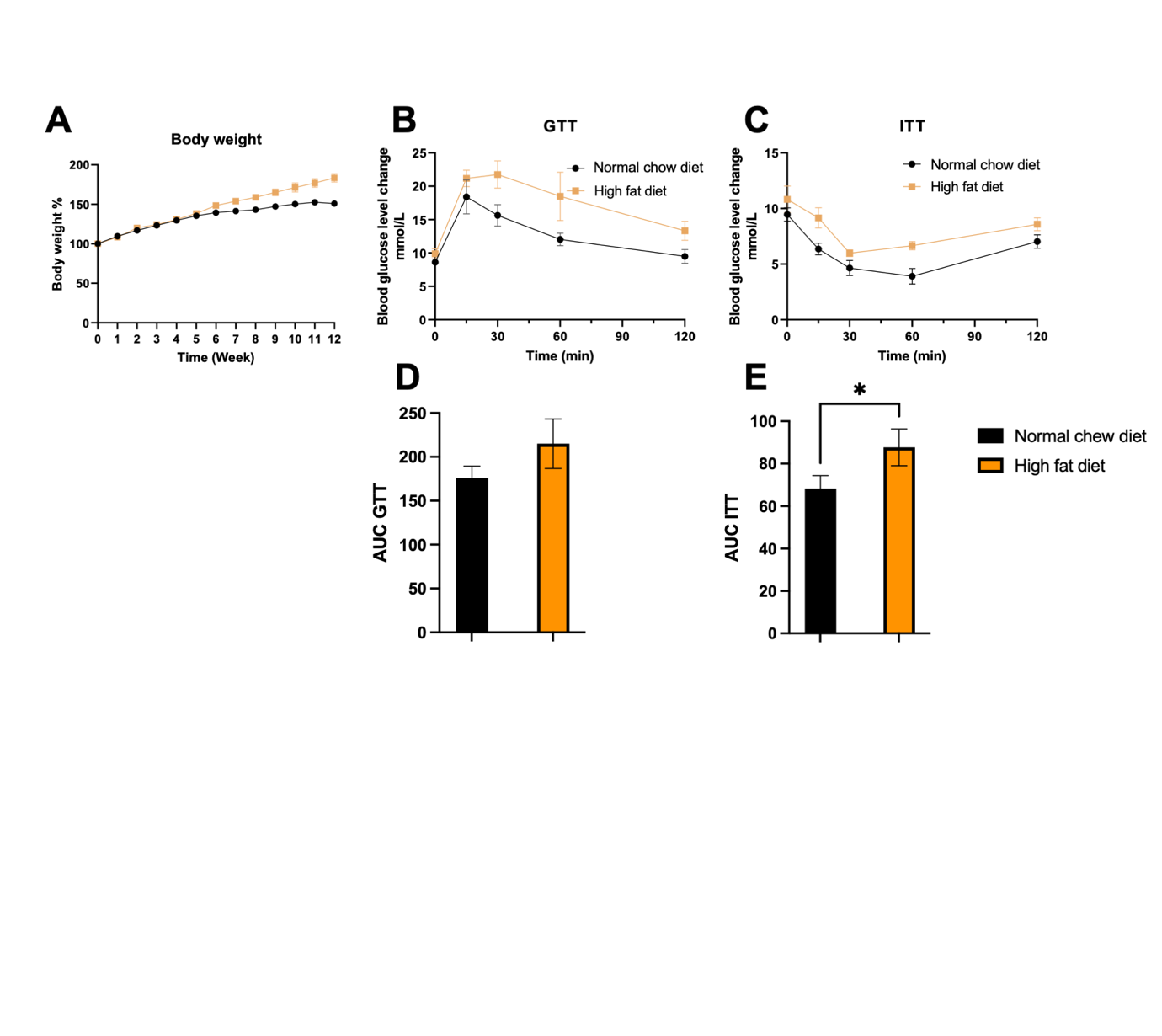


Figure S22 (A) The mice body weight changes with normal chow diet and high fat diet. The blood glucose level change after glucose tolerance test (GTT) (B) and insulin tolerance test (ITT) (C), and their area under the curve (AUC). n=3 (normal chow diet), n=5 (high fat diet), mean ± SD.

**Reference**

1. Abeer, M.M., et al., *Rationally designed dendritic silica nanoparticles for oral delivery of exenatide.* Pharmaceutics, 2019. **11**(8): p. 418.

2. Lamson, N.G., et al., *Anionic nanoparticles enable the oral delivery of proteins by enhancing intestinal permeability.* Nature biomedical engineering, 2020. **4**(1): p. 84–96.

3. McLenon, J. and M.A. Rogers, *The fear of needles: A systematic review and meta‐analysis.* Journal of advanced nursing, 2019. **75**(1): p. 30–42.

4. Lamson, N.G., et al., *Thrifty, rapid intestinal monolayers (TRIM) using caco-2 epithelial cells for oral drug delivery experiments.* Pharmaceutical Research, 2019. **36**: p. 1–12.

5. Janjua, T.I., et al., *Silica nanoparticles: A review of their safety and current strategies to overcome biological barriers.* Advanced Drug Delivery Reviews, 2023. **203**: p. 115115.
